# Dietary American Cockroach (*Periplaneta americana*) Chitin Influences Growth Performance, Nutrient Utilization, Gut Microbiota, and Riboflavin‐Related Metabolism in Juvenile Grass Carp (*Ctenopharyngodon idella*)

**DOI:** 10.1155/anu/3097646

**Published:** 2026-10-09

**Authors:** Xiaofang Zou, Qiujie Chen, Wei Pu, Jingjing Gao, Yongshou Yang, Xiaowen Long

**Affiliations:** ^1^ College of Agriculture and Biological Science Dali University Dali Yunnan China dali.edu.cn; ^2^ Cangshan Forest Ecosystem Observation and Research Station of Yunnan Province Dali Yunnan China; ^3^ Co-Innovation Center for Cangshan Mountain and Erhai Lake Integrated Protection and Green Development of Yunnan Province Dali University Dali Yunnan China dali.edu.cn; ^4^ National-Local Joint Engineering Research Center of Entomoceutics Dali University Dali Yunnan China dali.edu.cn

**Keywords:** *Ctenopharyngodon idella*, gut microbiota, nutrient digestibility, *Periplaneta americana* chitin, riboflavin-related metabolism

## Abstract

The American cockroach (*Periplaneta americana*) is an emerging sustainable source of insect‐based chitin, yet its physiological effects and optimal inclusion levels in herbivorous fish diets remain undefined. This study evaluated the dose‐dependent effects of dietary *P. americana* chitin (0%, 2.5%, 5%, 10%, and 20%) on juvenile grass carp (*Ctenopharyngodon idella*) over a 63‐day trial. Results revealed a distinct biphasic response in growth and nutrient utilization. Fish fed the 5% chitin diet exhibited the highest weight gain and specific growth rate (SGR), coinciding with the highest apparent digestibility coefficient (ADC) of crude protein (74.34%) compared with the control (72.01%). Conversely, higher inclusion levels (10% and 20%) significantly suppressed growth and reduced protein digestibility. Metagenomic reanalysis showed that the riboflavin‐biosynthesis gene ribA (K01497) was significantly reduced at 2.5%–10% dietary chitin (F (4,10) = 7.78, FDR *p* = 0.041), while the overall riboflavin metabolism pathway, the M00125 biosynthesis module, and intestinal riboflavin levels did not differ significantly among diets. Among the inclusion levels tested, 5% produced the most favorable overall response under the present experimental conditions, balancing improved growth and protein digestibility with the negative responses observed at higher inclusion levels.

## 1. Introduction

The American cockroach (*Periplaneta americana*) is widely distributed across tropical, subtropical, and temperate regions, characterized by its exceptional reproductive capacity, remarkable environmental adaptability, and diverse feeding habits [1, 2]. This species demonstrates significant efficiency in converting various organic waste materials into valuable biomass, positioning it as a promising sustainable feed resource for multiple animal species, thereby addressing feed shortages while mitigating environmental degradation [3, 4]. The nutritional profile of the American cockroach, particularly its high crude protein content, favorable amino acid composition comparable to fishmeal, and substantial fat content, has enabled its successful implementation as a partial or complete fishmeal replacement in diets for various aquatic species, including rainbow trout (*Oncorhynchus mykiss*) and juvenile common carp (*Cyprinus carpio*) [5–7].

Moreover, the American cockroach is rich in various bioactive compounds, such as antimicrobial peptides, polysaccharides, nucleosides, polyols, and chitin [3]. Chitin is a high‐quality raw material for promoting animal health and serves as a potential alternative to antibiotics, holding significant potential for further development and application [8]. Chitin, a linear polysaccharide composed of N‐acetylglucosamine units linked by *β*‐1,4‐glycosidic bonds, constitutes a fundamental structural component of insect exoskeletons [9]. Conventionally, industrial‐scale chitin is primarily derived from crustacean processing waste (including shrimp, prawns, crabs, and lobsters), with chitin content ranging from 15% to 40% [10]. In comparison, the American cockroach larvae contain 4.4% to 8.4% chitin, while adult specimens can yield up to 15% chitin [11]. Farmed American cockroaches represent a potentially sustainable alternative source of chitin and its derivatives due to their ease of cultivation, efficient harvesting, exceptional environmental adaptability, and inherent resistance to various pathogens [12].

Extensive research has demonstrated that chitin derived from diverse sources, such as shrimp and crabs, can enhance various physiological functions in fish, including growth performance, antioxidant capacity, and immune response [13–16]. Gaudioso et al. [17] also demonstrated the beneficial effects of chitin on fish growth and gut health. However, chitin is a complex structural polysaccharide that poses digestive challenges for many nonspecialist fish. Previous studies utilizing crustacean‐derived chitin have indicated that while low doses can be beneficial, high inclusion levels often act as an antinutritional factor [18, 19]. Crucially, this growth suppression is likely not merely a toxicity issue but may stem from the physical properties of the chitin polymer interfering with digestive physiology, potentially by impeding enzyme–substrate interactions. Thus, determining the optimal inclusion window is critical to balancing its functional benefits against these potential physical‐inhibitory effects.

The grass carp (*Ctenopharyngodon idella*), a freshwater species belonging to the order Cypriniformes, family Cyprinidae, and genus *Ctenopharyngodon*, has gained widespread popularity in aquaculture due to its rapid growth rate, delicate flesh texture, exceptional nutritional value, and economic affordability [20]. While chitin from crustaceans (shrimp and crab) has been extensively studied in aquaculture, information regarding insect‐derived chitin remains scarce. Insects like *P. americana* may yield chitin with distinct physicochemical properties compared to marine sources. Furthermore, the specific physiological response of herbivorous fish species, such as grass carp (*C. idella*), to this novel insect‐derived fiber is entirely unknown. To date, no studies have investigated the application of *P. americana* chitin as a feed additive in aquaculture, representing a significant gap in our understanding of sustainable insect‐based feed resources.

Therefore, this study was designed to test the hypothesis that dietary *P. americana* chitin exhibits a strong dose‐dependent effect in juvenile grass carp. We hypothesized that a low, optimal inclusion level would act as a functional additive, improving growth and intestinal health. Conversely, we hypothesized that high inclusion levels would exceed the fish’s digestive capacity, acting as an intestinal stressor that impairs intestinal integrity and disrupts microbial homeostasis. To test this, we comprehensively evaluated the effects of dietary *P. americana* chitin (0% to 20%) on growth performance, feed utilization, body composition, digestive enzyme activity, metabolic parameters, intestinal morphology, and gut microbiota composition in juvenile grass carp. These findings identify the most favorable inclusion level among those tested and characterize the adverse responses associated with excessive chitin inclusion, providing a practical basis for its potential application in sustainable aquaculture.

## 2. Materials and Methods

### 2.1. Ethics Statement

The experimental design and procedure were approved by the Animal Ethics Committee of Dali University (No. 2020‐PZ‐46). All procedures followed the Guidelines for the Care and Use of Laboratory Animals in China.

### 2.2. Experimental Diets

The American cockroach residue utilized in this study was sourced from a commercial breeding facility in Midu County, Dali Prefecture, where the insects were primarily reared on a diet consisting of cornmeal, pumpkin, and potatoes. Adult cockroaches were harvested after 4 months of cultivation, thoroughly cleaned, and dried at 80–90°C. The dried specimens were subsequently ground and passed through a 250 µm mesh sieve in preparation for chitin extraction. The extraction process began with oven‐drying the American cockroach residue at 65°C for 8 h to eliminate residual moisture, yielding a dry powder. Defatting was accomplished through petroleum ether immersion, wherein the powder was soaked for 24 h with hourly agitation until the supernatant became clear, indicating complete lipid removal. Subsequently, 100 g of the defatted sample was combined with 3000 mL of 4 M NaOH solution and heated at 95°C for 3 h to facilitate protein removal. The resulting precipitate was repeatedly washed with distilled water until the supernatant reached neutrality (pH 7.0), after which it was collected, dried at 105°C, and weighed. To remove inorganic salts, the remaining sample was treated with 0.5 M HCl (15 mL per gram of sample) and incubated at 60°C for 2 h. Following multiple washes with distilled water until neutrality was achieved, the precipitate was collected, dried at 105°C, and weighed, yielding chitin. For further purification, the chitin was treated with 10% hydrochloric acid (30 mL per gram) for 30 min, followed by decolorization with 0.5% sodium hypochlorite (15 mL per gram) at 80°C for 4 h. After repeated washing with distilled water until the wash solution reached neutrality (pH 7.0), the purified precipitate was collected and dried at 105°C. Chitin purity was determined by quantifying glucosamine (GlcN) after acid hydrolysis according to Šulc [21], with appropriate modifications. Briefly, the purified chitin was hydrolyzed with 6 M HCl at 110°C for 24 h, and the released GlcN was quantified by HPLC using a C18 column after FMOC derivatization. Chitin content was calculated from the GlcN concentration and expressed on a dry‐matter basis. The purified product contained 96.0% chitin on a dry‐matter basis. The extracted chitin was characterized by adding a drop of 0.03% iodine–potassium iodide solution and a drop of 1% sulfuric acid, which produced a purple‐brown coloration on a porcelain spot plate. Upon addition of several drops of 75% sulfuric acid, the color gradually dissipated, providing qualitative confirmation of chitin [22]. This color reaction was used only for qualitative identification and not for quantitative determination of chitin purity.

The base feed was formulated using fish meal, casein, and gelatin as the primary protein sources and fish oil and soybean oil as the primary fat sources. The purified *P. americana* chitin was incorporated into the base diet at concentrations of 0%, 2.50%, 5%, 10%, and 20%, resulting in five distinct dietary formulations, each containing equal nitrogen (30.00%) and lipid (5.00%) content. The dietary inclusion levels were selected with reference to previous studies evaluating chitin or chitosan across a broad range of inclusion levels in fish [18, 19]. Low‐to‐moderate inclusion levels were selected to evaluate the potential functional effects of dietary chitin, whereas higher inclusion levels were included to assess the upper tolerance range and potential antinutritional effects. Accordingly, the 2.5% and 5% diets represented low‐to‐moderate supplementation levels, while the 10% and 20% diets were used to evaluate responses to relatively high chitin inclusion; the 0% diet served as the control. This graded design also allowed the assessment of linear and quadratic dose–response relationships. For feed preparation, all ingredients were finely ground and passed through a 60‐mesh sieve. The components were thoroughly mixed according to the formulation proportions, with vitamins, minerals, and trace elements incorporated using a geometric dilution method. After the addition of soybean oil, fish oil, and water, the mixture was homogenized and processed through a pelletizer to form 1.5 mm diameter pellets (Table 1). The pellets were air‐dried at ambient temperature, sealed in airtight bags, and stored at −20°C until use.

**Table 1:** Formulation and proximate composition of diets.

Items	Diets				
	Diet 1	Diet 2	Diet 3	Diet 4	Diet 5
Ingredients (% dry matter)					
Fishmeal	10	10	10	10	10
PAC^a^	0	2.5	5	10	20
Soybean meal	10	10	10	10	10
Wheat flour	27.5	27.5	27.5	27.5	27.5
Casein	3.15	3.15	3.15	3.15	3.15
Rapeseed meal	10	10	10	10	10
Dextrin	20	17.5	15	10	0
Gelatin	4.5	4.5	4.5	4.5	4.5
Cottonseed meal	8	8	8	8	8
Fish oil	1	1	1	1	1
Soybean oil	1	1	1	1	1
Soy lecithin	1	1	1	1	1
Vitamin premix^b^	1	1	1	1	1
Mineral premix^c^	1	1	1	1	1
Vitamin C phosphate (30%)	0.05	0.05	0.05	0.05	0.05
Choline chloride	0.25	0.25	0.25	0.25	0.25
Antioxidants	0.05	0.05	0.05	0.05	0.05
Ca(H_2_PO_4_)_2_	1	1	1	1	1
Cr_2_O_3_	0.5	0.5	0.5	0.5	0.5
Total	100	100	100	100	100
Proximate composition (%)					
Moisture	10.51	10.20	10.06	10.30	10.70
Dry matter	89.49	89.80	89.94	89.70	89.30
Crude protein	30.46	30.43	30.38	30.41	30.41
Total lipid	5.32	5.38	5.98	5.55	5.22
Organic matter	80.73	81.45	82.36	81.55	80.59
Crude fiber	5.92	5.95	6.05	6.17	6.25

^a^PAC, *Periplaneta americana* chitin.

^b^Vitamin premix (per kg diet): vitamin A, 62,500 IU; vitamin D_3_, 15,000 IU; vitamin E, 1.75 g; vitamin K_3_, 35.4 mg; vitamin B_1_, 100 mg; vitamin B_2_, 150 mg; vitamin B_6_, 150 mg; vitamin B_12_, 0.2 mg; biotin, 4 mg; D‐calcium pantothenate, 250 mg; folic acid, 25 mg; nicotinamide, 300 mg; vitamin C, 700 mg.

^c^Mineral premix (per kg diet): FeSO_4_.H_2_O, 200 mg; CuSO_4_·5H_2_O, 96 mg; ZnSO_4_.H_2_O, 360 mg; MnSO_4_.H_2_O, 120 mg; MgSO_4_.H_2_O, 240 mg; KH_2_PO_4_, 4.2 g; NaH_2_PO_4_, 0.5 g; KI, 5.4 mg; CoCl_2_·6H_2_O, 2.1 mg; Na_2_SeO_3_, 3 mg.

### 2.3. Experimental Design and Rearing Management

The experiment was conducted at the Agricultural and Biological Science Laboratory of Dali University, Yunnan Province, China. A total of 225 juvenile *C. idella* with uniform size, free from external injuries, exhibiting good vitality, and with an initial average weight of ~20 g were selected for the study. Prior to experimental allocation, the fish were prophylactically treated by immersion in a 3% saline solution for 5 min. Subsequently, they were randomly distributed into 15 glass aquaria (dimensions: 0.9 m × 0.45 m × 0.6 m, length × width × height), each equipped with an independent filtration system. Following a 1‐week acclimation period during which fish were fed a basal diet, the formal experimental phase commenced.

The experimental design comprised five treatment groups with three replicates per treatment, each replicate containing 15 fish. Each aquarium was considered an independent experimental unit, resulting in three independent biological replicates per dietary treatment (*n* = 3). Fish within the same aquarium were considered subsamples rather than independent experimental replicates. Throughout the trial, daily feed intake (DFI), mortality, and water temperature were meticulously recorded for each experimental unit. Continuous aeration was provided via air pumps operating 24 h daily, and water quality parameters were monitored regularly based on fish behavior and condition. Water quality parameters, including pH, dissolved oxygen, nitrite, and ammonia concentrations, were measured every 2 days using a calibrated water quality analysis kit. Water conditions were maintained within optimal ranges for grass carp cultivation: water temperature was regulated between 28 and 30°C, dissolved oxygen concentration was maintained above 5.0 mg/L, pH stabilized between 7.0 and 8.5, ammonia concentration was kept below 0.5 mg/L, and nitrite concentration remained under 0.15 mg/L. Natural photoperiod was employed throughout the experimental duration. Feed was administered twice daily at 08:00 and 17:00 h. Following each 1 h feeding period, uneaten feed was carefully collected via siphoning. Daily feed allocation was calculated at 3%–5% of fish biomass. The breeding trial lasted 63 days.

### 2.4. Analyses of Growth Performance and Proximate Composition

Following the completion of the feeding experiment, fish were subjected to a 24 h fasting period. Survival was recorded for each experimental group. Five fish were randomly selected from each aquarium and anesthetized with eugenol (30 mg/L). After anesthetization, the fish were gently blotted with gauze to remove excess surface water. Body length and weight were measured, and internal organs and mesenteric fat were carefully dissected for subsequent growth index calculations. Growth and feed‐utilization indices were calculated separately for each aquarium using aquarium‐level initial and final biomass, mortality, and feed‐intake data. For body indices measured in the five sampled fish, individual measurements were averaged within each aquarium to generate one aquarium‐level value for subsequent statistical analysis. The following formulas were employed to calculate the survival rate (SR), weight gain rate (WGR), specific growth rate (SGR), DFI, feed conversion ratio (FCR), protein efficiency ratio (PER), condition factor (CF), viscerosomatic index (VSI), and hepatosomatic index (HSI):

SR % =100×Nt/N0,

WGR % =100×Wt−W0/ W0, 

SGR %/d=100×lnWt−lnW0/t,

DFI % =2F/T×Wi+Wh+Wd,

FCR=Wf/Wt−W0,

PER % =100×Wt−W0/Wf×Wp,

CF g/cm3% =100×Wt/ L3,

VSI % =100×Wv/ Wt,

HSI % =100×Wl/Wt,

where *N*_*t*_ is the final number of fish, *N*_0_ is the initial number of fish, *W*_0_ is the initial average body weight, *W*_*t*_ is the final average body weight, *t* is the experimental duration in days, *F* is the feed fed, *T* is the culture time, *W*_i_ is the initial body weight of fish, *W*_h_ is the final body weight (FBW) of fish, *W*_d_ is the weight of dead fish, *W*_f_ is the total feed intake, *W*_p_ is the crude protein content of the feed, *L* is the final average body length, *W*_v_ is the average viscera weight at the end of the experiment, and *W*_l_ is the average liver weight at the end of the experiment.

For each aquarium, blood samples were collected from five fish via caudal venipuncture using sterile syringes. The collected blood was stored at 4°C for 4 h, then centrifuged at 684 ×*g* for 5 min at 4°C. The resulting serum was separated, collected, and stored at −40°C for subsequent metabolic analyses. The five fish sampled from each aquarium were treated as subsamples nested within that aquarium.

Following blood collection, the fish were dissected under sterile conditions on ice. The liver, intestine, and esophagus samples were harvested from each replicate. After removing intestinal adipose tissue, hindgut contents were gently expressed using sterile forceps, immediately transferred to 1.5 mL sterile cryovials, and flash‐frozen in liquid nitrogen. These samples were stored at −80°C for gut microbiome and metabolomic analyses.

Segments of the foregut, midgut, and hindgut (0.5–1.0 cm each) were excised, thoroughly rinsed with sterile saline, fixed in 1 mL of 4% paraformaldehyde solution in 1.5 mL microcentrifuge tubes, and maintained at room temperature for subsequent histological examination. Additionally, ~1 cm segments of intestinal tissue were preserved in 1 mL of RNA later solution in 1.5 mL microcentrifuge tubes for digestive enzyme gene expression analysis. The remaining intestinal, hepatic, and esophageal tissues were sealed in sterile bags and stored at −40°C for digestive enzyme activity assays.

The proximate composition (moisture, crude protein, total fat, and ash content) of the experimental feeds, and whole fish samples were determined according to the standard methods of the AOAC [23]. Specifically, crude protein content was determined using the Kjeldahl method, crude fat content was measured by Soxhlet extraction with petroleum ether as the solvent, crude ash content was quantified by incineration at 550°C for 8 h in a muffle furnace, and moisture content was assessed by constant‐weight drying at 105°C.

### 2.5. Apparent Digestibility of Feed Nutrients

One hour after each feeding event, uneaten feed and fish excretions were carefully removed from the tanks. Fresh fecal matter that had settled at the bottom of the aquaria was collected twice daily (12:00 and 21:00) using a gentle siphoning technique. The collected feces were drained to eliminate excess water and immediately stored at −20°C. Feces were collected continuously for 28 days during the final stage of the feeding trial.

For analytical preparation, the fecal samples were dried at 70°C to constant weight, finely ground through a 40‐mesh sieve, and stored at −20°C pending nutritional composition analysis. The determination of crude protein and crude lipid content in both experimental feeds and fecal samples was conducted according to the standard methods established by AOAC [23]. Chitin content in both feed and fecal samples was assessed following the procedure described by Shahidi [24]. Chromium concentrations (Cr_2_O_3_), used as an inert marker, were measured using spectrophotometric analysis [25].

The apparent digestibility coefficients (ADCs) for crude protein, crude lipid, and chitin in the experimental diets were calculated according to the standardized methodology for aquatic animals [26].

ADCd=100×1−Nf/Nd×Md/Mf.

ADC_d_ represents the apparent digestibility of nutrients, expressed as a percentage (%). *M*_d_ refers to the percentage of the marker in the feed, expressed as a percentage (%). *M*_f_ indicates the percentage of the marker in the feces, expressed as a percentage (%). *N*_d_ is the percentage of the nutrient in the feed, expressed as a percentage (%). *N*_f_ represents the percentage of the nutrient in the feces, expressed as a percentage (%).

### 2.6. Biochemical Parameters Analysis

Approximately 0.3 g of liver, intestinal, or esophageal tissue was homogenized in 2.7 mL of cold 0.85% physiological saline solution using an IKA homogenizer (IKA T10B, Germany) at 10,000 r/min for 1 min. The resulting 10% homogenate was then centrifuged at 12,000 × *g* at 4°C for 10 min using a low‐temperature centrifuge (5417R, Eppendorf, Hamburg, Germany). The supernatant was collected and stored at −40°C for subsequent analysis.

The following biochemical parameters were measured in the tissue homogenate supernatant and serum using a spectrophotometer (T6, Beijing Pukenye General Instrument Co., Ltd., Beijing, China) and commercially available assay kits (Nanjing Jiancheng Bioengineering Institute, Nanjing, China): (a) Digestive enzymes from the liver, intestine, and esophagus, including trypsin (Cat. A080‐2), lipase (Cat. A054‐1), α‐amylase (Cat. C016‐1), cellulase (Cat. A138), chitinase (Cat. A139‐1), lactase (Cat. A082‐1), sucrase (Cat. A082‐2), and maltase (Cat. A082‐3); (b) Hepatic metabolic markers, including aspartate aminotransferase (AST, Cat. C010‐1), alanine aminotransferase (ALT, Cat. C009‐1), blood urea nitrogen (BUN, Cat. C013‐2), total cholesterol (TC, Cat. A111‐2), triglycerides (TG, Cat. A110‐2), glycogen (Cat. A043‐1), and glucose (GLU, Cat. F006‐1); (c) Serum metabolic markers, including total soluble protein (TP, Cat. A045‐2), aspartate aminotransferase (AST, Cat. C010‐1), alanine aminotransferase (ALT, Cat. C009‐1), blood urea nitrogen (BUN, Cat. C013‐2), total cholesterol (TC, Cat. A111‐2), triglycerides (TG, Cat. A110‐2), low‐density lipoprotein cholesterol (LDL‐C, Cat. A113‐2), high‐density lipoprotein cholesterol (HDL‐C, Cat. A112‐1), and glucose (GLU, Cat. F006‐1). For biochemical parameters and digestive‐enzyme activities, measurements obtained from the five fish sampled from each aquarium were averaged to generate one aquarium‐level value. The resulting three aquarium‐level values per dietary treatment were used as independent biological replicates for statistical analysis (*n* = 3).

### 2.7. Gene Expression Levels of Intestinal Digestive Enzymes

Total RNA was extracted from intestinal tissues using a Fast RNA Extraction Kit (Sangon Biotech, Shanghai, China). RNA concentration and purity were assessed using a microvolume UV–Vis spectrophotometer (Thermo Fisher Scientific, Shanghai, China). The A260/A280 and A260/A230 ratios ranged from 1.8 to 2.2 and from 1.91 to 2.18, respectively, indicating acceptable RNA purity. Before reverse transcription, residual genomic DNA was removed using the gDNA digester included in the MightyScript Plus First Strand cDNA Synthesis Master Mix (Sangon Biotech, Shanghai, China; Cat. No. B639252) according to the manufacturer’s instructions. The treated RNA was subsequently reverse transcribed into cDNA using the same kit.

Primers for intestinal digestive enzyme genes were designed using the primer design tool available on the NCBI website. Gene expression analysis was performed via RT‐qPCR using the CFX Duet System (Bio‐Rad Laboratories, Inc., USA) with 2x HyperMB Universal SYBR Green qPCR Master Mix according to the manufacturer’s protocol. Each 20 μL reaction contained 2 μL of cDNA template, 1 μL each of forward and reverse primers, 6 μL of nuclease‐free water, and 10 μL of 2x Universal SYBR Green mix. The RT‐qPCR procedure followed the methodology described by Cardinaletti et al. [27]. *β*‐actin served as the internal reference gene for normalization. Consistent with the aquarium‐based statistical framework, technical replicates were first averaged within each fish, and fish‐level Cq values were then averaged within each aquarium. The variation and stability assessment of *β*‐actin Cq values among dietary treatments are presented in Supporting Information 1: Table S1. *β*‐actin Cq values differed significantly among dietary treatments (one‐way ANOVA, F(4,10) = 8.21, *p* = 0.003), with an overall CV of 4.79% and within‐group CVs of 0.8%–4.0%. Because this significant between‐diet shift indicates that *β*‐actin was not invariant across treatments, the use of a single reference gene is acknowledged as a limitation. As a sensitivity analysis, raw (non‐normalized) target‐gene Cq values were analyzed at the aquarium level; the principal treatment effects remained significant for chitinase (*p* = 0.016), maltase (*p* = 0.006), trypsin (*p* = 0.033), and *α*‐amylase (*p* = 0.026). These raw‐Cq analyses provide a sensitivity check on the principal treatment effects but do not constitute independent evidence of reference‐gene stability. Subsequently, the relative expression levels of trypsin, lipase, *α*‐amylase, chitinase, sucrase, maltase, and lactase were calculated using the 2^−ΔΔCq^ method. The primer sequences for both the reference gene and target genes used in RT‐qPCR analysis are presented in Table 2.

**Table 2:** Nucleotide sequence of primers used to assay gene expression for real‐time quantitative polymerase chain reaction (RT‐qPCR).

Target gene	Forward (5’ to 3’)	Reverse (5’ to 3’)	Accession number
*β*‐actin	CGTGCTGTCTTCCCATCCATCG	CTGAGCCTCGTCACCAACATAGC	XM_051886219.1
Trypsin	AGCCTCTGGGCTGTGTCTGC	TGCTCGGTGCCCTCAGTGAC	XM_051864373
Lipase	GGCACGGAAGAGGATACAAGCAAG	CTGTCAGGACACTGTCGCAGAATG	XM_051872146
*α*‐amylase	TTCAACGCCAACAAGGAGGACTTC	CAGGAGACTCACCAAGCGACAATC	XM_051867608
Chitinase	TGCGTATTTGGCGTGCTGTCC	ACCACCCCATCACCTCACTCTTG	XM_051880285
Lactase	GCTACACTGCCTGGTCACTAATGG	GCGGTAAAGCGGGATCTGATCG	XM_051890193
Sucrase	TGGCGGTCTGGACTGTCTATCTG	GGAAGCGTTGGGATCTGGGAAAC	XM_051888976
Maltase	ACCAATGCAACAGTCACACCAGAG	AGTAGCATCAGGTGAGGTCGTCTC	XM_051893102

### 2.8. Histological Analysis of the Foregut, Midgut, and Hindgut

Tissue samples were fixed in 4% paraformaldehyde, rinsed with water, and then dehydrated through a graded ethanol series. Subsequently, samples were cleared with xylene, embedded in paraffin, and sectioned at a thickness of 5 µm. Hematoxylin‐eosin (HE) staining was performed on the sections, which were then sealed with neutral gum. After drying, an Olympus microscope was used to observe and photograph the crypt depth, villus length, and muscle thickness. The resulting histological images were subsequently analyzed and processed using K‐Viewer v1.5.3 software.

### 2.9. Metabolomic Analysis of Intestinal Contents

Approximately 200 mg of intestinal‐content sample was accurately weighed into a 2 mL centrifuge tube, after which 600 μL of methanol containing 2‐chloro‐L‐phenylalanine at 4 μg/mL was added. The mixture was vortexed for 30 s using a vortex mixer (BE‐2600, Haimen Kylin‐bell Lab Instruments Co., Ltd., Haimen, China). Steel beads were added, and the sample was ground using a tissue grinder (MB‐96, Zhejiang Meibi Experiment Equipment Co., Ltd., Zhejiang, China) at 50 Hz for 120 s. Subsequently, the mixture was sonicated at room temperature for 10 min (KQ‐800DE, Kunshan Shumei Experiment Equipment Co., Ltd., Kunshan, China). The homogenate was then centrifuged at 12,000 × *g* for 10 min at 4°C (H1850‐R, Hunan Xiangyi Experiment Equipment Co., Ltd., Hunan, China). The supernatant was collected and filtered through a 0.22 μm PTFE membrane (Tianjin Jinteng Experiment Equipment Co., Ltd., Tianjin, China). Finally, the filtrate was transferred to a vial for LC‐MS analysis. In metabolomics studies based on mass spectrometry, XCMS software was used to extract metabolite ion peaks in both positive and negative ion modes to ensure reliable and high‐quality metabolomic data for analysis and quality control [28, 29]. Orthogonal partial least squares discriminant analysis (OPLS‐DA) was performed on the sample data using R/Ropls (V1.22.0), and overfitting was assessed through permutation testing. A heatmap of differentially expressed metabolites was generated using R/pheatmap (V1.0.12). KEGG pathway enrichment analysis of the differential metabolites was performed using a hypergeometric test in R (v4.0.3). Identified metabolites were annotated using the KEGG database and mapped to the KEGG pathway database. Differential metabolites were identified based on a variable importance in projection (VIP) >1.0 and a *t*‐test *p*‐value < 0.05. Spearman correlation coefficients between significantly altered gut microbiota and differential metabolites were calculated in R, and the resulting correlation matrix was visualized using the pheatmap package (v1.0.12).

### 2.10. 16S rRNA Amplicon Sequencing and Community Structure Analysis

To investigate the gut microbial community, this study employed a complementary multiomics approach. The 16S rRNA amplicon sequencing was utilized to provide a high‐depth analysis of taxonomic composition and community diversity. The hypervariable V3–V4 region of the bacterial 16S rRNA gene was amplified using universal primers (341 F: 5^′^‐CCTAYGGGRBGCASCAG‐3^′^ and 806R: 5^′^‐GGACTACNNGGGTATCTAAT‐3^′^). PCR amplicons were purified, and libraries were prepared using the NEBNext Ultra DNA Library Prep Kit for Illumina (NEB, USA, Catalog No. E7370L) according to the manufacturer’s protocol, with unique index codes assigned to each sample. Library quality was evaluated on the Agilent 5400 system. Qualified libraries were pooled and sequenced on an Illumina NovaSeq platform using the PE150 strategy.

Raw sequencing reads were processed using the QIIME 2 pipeline (v2022.2). Briefly, raw paired‐end reads were quality‐filtered, denoised, merged, and chimera‐removed using the DADA2 plugin to generate amplicon sequence variants (ASVs). Taxonomic classification was assigned to ASVs using a pre‐trained Naive Bayes classifier against the SILVA (v138) 16S rRNA database. Alpha diversity indices (including Chao1, Shannon, Simpson, observed features, and Faith’s phylogenetic diversity (Faith PD)) and beta diversity (based on Bray–Curtis dissimilarity and weighted/unweighted UniFrac) were calculated. Principal coordinates analysis (PCoA) was used to visualize the separation of microbial communities. This amplicon sequencing approach was employed to provide a robust and high‐resolution analysis of taxonomic composition and community diversity, and these taxonomic results were specifically utilized for the correlation analysis against differential gut metabolites.

### 2.11. Shotgun Metagenomic Sequencing and Functional Profile Analysis

To complement the deep taxonomic profiling derived from 16S rRNA sequencing, shotgun metagenomic sequencing was performed with the primary objective of characterizing the functional potential and gene family abundance of the gut microbiome. This strategy leverages the strengths of each method: 16S sequencing for robust taxonomic classification and diversity analysis, and shotgun metagenomics for comprehensive functional profiling. Genomic DNA was extracted from the intestinal contents of juvenile grass carp using the CTAB method. Sequencing libraries were constructed using the NEBNext Ultra DNA Library Prep Kit for Illumina (NEB, USA, Catalog No. E7370L) according to the manufacturer’s protocol, with unique index codes assigned to each sample. Library quality was evaluated on the Agilent 5400 system and quantified by qPCR to a final concentration of 1.5 nM. Qualified libraries were pooled and sequenced on Illumina platforms using the PE150 strategy, based on the required effective library concentration and data output. Metagenomic sequencing was performed on the Illumina NovaSeq high‐throughput platform.

To analyze the functional potential, raw sequencing reads were processed using a standard pipeline. First, KneadData software was used for quality control (e.g., trimming low‐quality bases) and filtering host‐derived reads (against the *Ctenopharyngodon idella* genome). The resulting high‐quality, nonhost reads were then processed using HUMAnN3 (v3.6) [30]. This pipeline utilized DIAMOND to align reads against the UniProt UniRef90 protein database to quantify gene family abundance, which was normalized as copies per million (CPM). Functional pathway abundance was determined by mapping gene families to the KEGG (Kyoto Encyclopedia of Genes and Genomes) database. To evaluate the riboflavin‐biosynthesis hypothesis prompted by the metabolomic results, KEGG Orthology (KO) abundances for the riboflavin metabolism pathway (ko00740), the bacterial riboflavin‐biosynthesis module (M00125), and the genes ribA (K01497), ribB (K02858), ribD (K11752), ribE (K00793), ribH (K00794), ribF (K11753), and ribBA (K14652) were extracted from the HUMAnN unstratified KO table. Pathway, module, and gene abundances were compared among diets by one‐way ANOVA followed by Benjamini–Hochberg FDR correction across the 10 tested features. Spearman rank correlation (tank level, *n* = 15) was used to relate *Cetobacterium* relative abundance (16S rRNA amplicon) to the riboflavin‐biosynthesis module. Taxon‐stratified contributions were inspected in the HUMAnN stratified KO table.

To further evaluate the potential functional relationship between the gut microbiota and riboflavin‐related metabolism, a targeted reanalysis of the metagenomic dataset was performed. KEGG Orthology genes assigned to the riboflavin metabolism pathway (ko00740) and the bacterial riboflavin‐biosynthesis module (M00125) were extracted from the HUMAnN functional‐profile output. The analysis included the principal bacterial riboflavin‐biosynthesis genes *ribA*, *ribB*, *ribD*, *ribE*, and *ribH*, together with genes involved in the conversion of riboflavin into flavin mononucleotide and flavin adenine dinucleotide. The aggregate abundance of the riboflavin‐biosynthesis module and the abundances of individual functional genes were compared among dietary groups. Spearman’s correlation analysis was used to examine the association between the relative abundance of *Cetobacterium* and microbial riboflavin‐biosynthesis potential. Where supported by the taxon‐stratified HUMAnN output, the contribution of *Cetobacterium*‐associated sequences to the annotated riboflavin‐biosynthesis pathway was also evaluated.

### 2.12. Statistical Analysis

All data are presented as mean ± SE (*n* = 3). Each aquarium was considered an independent experimental unit because dietary treatments were administered at the aquarium level. Each dietary treatment consisted of three independent aquaria. For variables measured in individual fish, the five fish sampled from each aquarium were treated as subsamples, and their measurements were averaged to generate one aquarium‐level value. Therefore, statistical analyses were performed using three independent aquarium‐level replicates per dietary treatment (*n* = 3), thereby avoiding pseudoreplication of fish maintained within the same aquarium. All statistical analyses were performed using the SPSS statistical software (version 21.0). The homogeneity of variance was tested with Levene’s test. When necessary, arcsine‐square root or logarithmic transformation was performed before analysis. The differences among the five diet groups were statistically analyzed using one‐way analysis of variance (ANOVA) and compared with the Duncan’s multiple range test. *p*  < 0.05 was considered statistically significant. To specifically evaluate the dose‐dependent effects of dietary *P. americana* chitin, orthogonal polynomial contrasts were used to determine the linear and quadratic responses to the graded inclusion levels. For the targeted metagenomic analysis of riboflavin‐related functions, pathway‐, module‐, and gene‐level abundances were compared among dietary groups using one‐way ANOVA, followed by Benjamini–Hochberg false discovery rate (FDR) correction across the tested features. When a significant overall treatment effect was detected for an individual gene, Tukey’s honestly significant difference (HSD) test was used for post hoc pairwise comparisons. For gut microbial alpha‐diversity indices, the overall differences among dietary groups were evaluated using the Kruskal–Wallis test.

## 3. Results

### 3.1. SRs and Growth Performance

Dietary supplementation with *P. americana* chitin had no significant effect on the SR of juvenile grass carp (*p* > 0.05, Table 3). However, growth parameters, including FBW, WGR, and SGR, exhibited a significant quadratic response to chitin inclusion (*p*  < 0.05). These indices improved with increasing chitin levels, peaking in the 5% group (Diet 3), before significantly declining at inclusion levels of 10% and 20%. Feed utilization followed a similar optimal trend; the 5% group achieved the lowest FCR and the highest PER. Conversely, high inclusion levels (10% and 20%) significantly suppressed DFI compared to the control and lower‐dose groups. The VSI was significantly highest in the 20% group (Diet 5; *p*  < 0.05), while the 5% group (Diet 3) had the lowest HSI. No significant differences were observed in the CF among the dietary groups.

**Table 3:** Survival rates and growth performance of juvenile *Ctenopharyngodon idella*.

Items	Diets					*p*‐Value		
	Diet 1	Diet 2	Diet 3	Diet 4	Diet 5	ANOVA	Linear	Quadratic
SR (%)	96.67 ± 3.34	93.34 ± 6.67	86.67 ± 6.67	91.11 ± 2.22	90.00 ± 3.34	0.638	0.437	0.530
IBW (g)	20.09 ± 0.02	20.16 ± 0.02	20.09 ± 0.03	20.15 ± 0.03	20.15 ± 0.03	0.240	0.225	0.441
FBW (g)	34.24 ± 0.36^bc^	36.11 ± 1.58^ab^	38.34 ± 1.02^a^	31.73 ± 0.48^cd^	30.72 ± 0.79^d^	0.001	0.011	0.034
WGR (%)	70.43 ± 1.89^bc^	79.14 ± 7.65^ab^	90.83 ± 5.07^a^	57.45 ± 2.22^cd^	52.45 ± 3.96^d^	0.001	0.010	0.032
SGR (%/d)	0.84 ± 0.02^bc^	0.92 ± 0.07^ab^	1.02 ± 0.04^a^	0.72 ± 0.02^cd^	0.67 ± 0.04^d^	0.001	0.007	0.025
DFI (%)	1.31 ± 0.11^a^	1.39 ± 0.02^a^	1.38 ± 0.03^a^	1.11 ± 0.04^b^	1.00 ± 0.06^b^	0.002	<0.001	0.002
FCR	1.85 ± 0.09^ab^	1.75 ± 0.05^b^	1.62 ± 0.01^b^	1.70 ± 0.08^b^	2.22 ± 0.23^a^	0.039	0.030	0.004
PER	1.79 ± 0.09^ab^	1.89 ± 0.05^a^	2.03 ± 0.01^a^	1.94 ± 0.09^a^	1.52 ± 0.16^b^	0.025	0.045	0.003
CF (g/cm^3^)	1.88 ± 0.07	1.84 ± 0.02	1.83 ± 0.04	1.92 ± 0.06	2.07 ± 0.23	0.599	0.109	0.245
VSI (%)	13.29 ± 0.76^ab^	11.75 ± 0.51^b^	11.35 ± 0.71^b^	12.25 ± 0.74^b^	14.87 ± 0.88^a^	0.041	0.049	0.010
HSI (%)	2.44 ± 0.25^a^	2.21 ± 0.06^ab^	1.68 ± 0.12^b^	1.92 ± 0.20^ab^	2.08 ± 0.09^ab^	0.047	0.572	0.045

*Note*: Data are presented as mean ± SE (*n* = 3). Values within the same row with different lowercase letters differ significantly (*p*  < 0.05). Linear and quadratic dose–response relationships were evaluated using orthogonal polynomial contrasts.

Abbreviations: CF, condition factor; DFI, daily feed intake; FBW, final body weight; FCR, feed conversion ratio; HSI, hepatosomatic index; IBW, initial body weight; PER, protein efficiency ratio; SGR, specific growth rate; SR, survival rates; VSI, viscerosomatic index; WGR, weight gain rate.

### 3.2. Apparent Digestibility of Nutritional Components in the Experimental Diets

A strong dose‐dependent effect was observed in nutrient digestibility (Table 4). The ADC of crude protein displayed a significant quadratic trend (*p*  < 0.05), reaching its maximum in the 5% group, which was significantly higher than the control. However, inclusion levels of 10% and 20% resulted in a sharp, significant reduction in protein ADC, falling below control levels. Chitin digestibility followed a similar pattern, being highest in the 2.5% and 5% groups and significantly decreasing at 10% and 20% inclusion. No significant differences were observed in the ADC of crude lipid among the dietary groups. Regression analyses confirmed significant linear and quadratic correlations for crude protein and chitin digestibility (*p*  < 0.05).

**Table 4:** Effect of *Periplaneta americana* chitin on the apparent digestibility of the nutritional components of juvenile *Ctenopharyngodon idella*.

Items	Diets					*p*‐Value		
	Diet 1	Diet 2	Diet 3	Diet 4	Diet 5	ANOVA	Linear	Quadratic
Crude protein (%)	72.01 ± 0.21^b^	73.94 ± 0.16^ab^	74.34 ± 1.02^a^	66.78 ± 0.00^c^	60.76 ± 0.79^d^	<0.001	<0.001	0.001
Total lipid (%)	81.55 ± 1.20	77.42 ± 4.43	80.98 ± 1.88	79.23 ± 1.38	77.84 ± 1.86	0.701	0.418	0.737
Chitin (%)	80.27 ± 6.93^ab^	88.97 ± 3.31^a^	86.87 ± 2.23^a^	72.53 ± 1.06^b^	67.35 ± 0.95^b^	0.033	0.012	0.048

*Note*: Data are presented as mean ± SE (*n* = 3). Values within the same row with different lowercase letters differ significantly (*p*  < 0.05). Linear and quadratic dose–response relationships were evaluated using orthogonal polynomial contrasts.

### 3.3. Proximate Composition in Juvenile Grass Carp Body

Dietary chitin supplementation did not significantly alter the whole‐body proximate composition of juvenile grass carp. Moisture, crude protein, crude lipid, and ash contents remained statistically similar across all treatment groups (*p* > 0.05, Table 5).

**Table 5:** Proximate composition of whole‐body of juvenile *Ctenopharyngodon idella*.

Items	Diets					*p*‐Value		
	Diet 1	Diet 2	Diet 3	Diet 4	Diet 5	ANOVA	Linear	Quadratic
Moisture (%)	74.61 ± 0.64	74.25 ± 0.29	74.80 ± 0.32	75.26 ± 0.49	75.10 ± 0.57	0.227	0.403	0.621
Crude protein (%)	15.61 ± 0.90	15.60 ± 0.55	15.89 ± 0.50	15.07 ± 0.15	15.69 ± 0.33	0.856	0.481	0.766
Total lipid (%)	4.82 ± 0.29	5.01 ± 0.12	4.81 ± 0.16	4.90 ± 0.19	4.81 ± 0.26	0.843	0.950	0.944
Ash (%)	2.47 ± 0.08	2.43 ± 0.14	2.44 ± 0.06	2.67 ± 0.19	2.59 ± 0.07	0.235	0.434	0.596

*Note*: Data are presented as mean ± SE (*n* = 3). Values within the same row with different lowercase letters differ significantly (*p*  < 0.05). Linear and quadratic dose–response relationships were evaluated using orthogonal polynomial contrasts.

### 3.4. Metabolic Parameters in the Serum and Liver

In the serum, high‐dose chitin supplementation (10% and/or 20%) induced signs of metabolic stress and lipid mobilization (Table 6). Specifically, the 20% group exhibited significantly elevated AST activity and glucose (GLU) levels compared to the optimal 5% group. Serum triglycerides (TG) were also significantly higher in the 10% and 20% groups. In the liver, a shift in energy storage was observed; glycogen (GLY) content significantly increased in the high‐dose groups (10% and 20%), whereas hepatic TG levels were lowest in the 20% group (*p*  < 0.05). Liver AST activity showed a significant reduction in the 10% group compared to the control.

**Table 6:** Metabolic indices in the serum and liver of juvenile *Ctenopharyngodon idella*.

Items	Diets					*p*‐Value		
	Diet 1	Diet 2	Diet 3	Diet 4	Diet 5	ANOVA	Linear	Quadratic
Serum								
TP (g/L)	18.53 ± 0.73	22.10 ± 2.04	20.01 ± 0.19	19.81 ± 2.35	20.87 ± 1.30	0.590	0.659	0.900
AST (U/L)	23.75 ± 7.56^ab^	20.00 ± 3.07^ab^	6.84 ± 1.61^b^	11.50 ± 4.50^ab^	25.99 ± 7.68^a^	0.036	0.823	0.007
ALT (U/L)	15.09 ± 0.97	15.84 ± 0.08	14.15 ± 0.08	15.30 ± 1.66	15.05 ± 1.08	0.816	0.931	0.978
BUN (mmol/L)	4.18 ± 0.10	4.07 ± 0.61	3.48 ± 0.20	3.77 ± 0.81	3.93 ± 0.62	0.899	0.819	0.717
TC (mmol/L)	6.91 ± 0.53	7.59 ± 0.35	7.15 ± 1.29	7.95 ± 0.83	6.46 ± 1.43	0.837	0.631	0.550
TG (mmol/L)	2.60 ± 0.06^ab^	2.26 ± 0.28^ab^	1.71 ± 0.18^b^	3.01 ± 0.71^a^	3.13 ± 0.33^a^	0.034	0.177	0.011
LDL‐C (mmol/L)	0.43 ± 0.27	0.48 ± 0.11	0.54 ± 0.22	0.28 ± 0.08	0.35 ± 0.07	0.837	0.475	0.772
HDL‐C (mmol/L)	1.95 ± 0.54	2.40 ± 0.16	2.72 ± 0.26	2.17 ± 0.27	2.35 ± 0.75	0.803	0.841	0.858
GLU (mmol/L)	3.27 ± 0.36^b^	3.95 ± 0.37^ab^	5.07 ± 0.41^ab^	4.87 ± 1.14^ab^	5.73 ± 0.71^a^	0.032	0.014	0.033
Liver								
AST (U/g protein)	5.82 ± 1.06^a^	5.43 ± 1.00^a^	5.11 ± 1.21^ab^	1.72 ± 0.54^b^	3.60 ± 1.46^ab^	0.006	0.007	0.001
ALT (U/g protein)	4.48 ± 1.03	4.41 ± 0.32	5.35 ± 0.57	4.49 ± 0.10	4.96 ± 0.18	0.709	0.621	0.874
BUN (mmol/g protein)	1.48 ± 0.29	1.41 ± 0.10	1.30 ± 0.10	1.23 ± 0.21	1.31 ± 0.02	0.858	0.444	0.490
TC (mmol/g tissue)	4.55 ± 0.51	4.15 ± 0.20	4.95 ± 0.20	4.66 ± 0.06	4.37 ± 0.11	0.341	0.846	0.600
TG (mmo/g tissue)	27.30 ± 2.12^a^	31.06 ± 2.13^a^	24.34 ± 3.76^ab^	24.09 ± 1.98^ab^	17.63 ± 1.85^b^	0.034	0.003	0.016
GLY (mg/g tissue)	18.63 ± 0.94^a^	19.20 ± 1.54^a^	21.18 ± 2.57^a^	34.64 ± 1.73^b^	33.97 ± 4.34^b^	0.001	0.001	0.001
GLU (mmol/g protein)	1.76 ± 0.10	1.66 ± 0.05	1.65 ± 0.03	1.65 ± 0.04	1.74 ± 0.05	0.620	0.762	0.297

*Note*: Data are presented as mean ± SE (*n* = 3). Values within the same row with different lowercase letters differ significantly (*p*  < 0.05). Linear and quadratic dose–response relationships were evaluated using orthogonal polynomial contrasts.

Abbreviations: ALT, alanine aminotransferase; AST, aspartate aminotransferase; BUN, blood urea nitrogen; GLU, glucose; GLY, glycogen; HDL‐C, high‐density lipoprotein cholesterol; LDL‐C, low‐density lipoprotein cholesterol; TC, total cholesterol; TG, triacylglycerol; TP, total protein.

### 3.5. Digestive Enzyme Activity in the Liver, Intestine, and Esophagus

Digestive enzyme activities exhibited tissue‐specific responses (Table 7). In the liver and intestine, the activities of major proteolytic and amylolytic enzymes (trypsin and *α*‐amylase) remained statistically unaffected by dietary chitin (*p* > 0.05). However, specific responses were noted in other enzymes: hepatic maltase showed a quadratic increase, peaking in the 20% group, while intestinal lipase and sucrase activities were highest in the low‐to‐moderate inclusion groups (2.5% and 5%) before declining at higher doses. In the esophagus, lipase activity significantly decreased with increasing chitin levels, while chitinase activity was highest in the 20% group.

**Table 7:** Digestive enzyme activities in the liver intestine and esophagus of juvenile *Ctenopharyngodon idella*.

Items	Diets					*p*‐Value		
	Diet 1	Diet 2	Diet 3	Diet 4	Diet 5	ANOVA	Linear	Quadratic
Liver								
Trypsin (U/mg protein)	3666.43 ± 859.01	2988.26 ± 259.69	3536.54 ± 357.34	2905.16 ± 184.54	3218.45 ± 201.74	0.704	0.587	0.652
Lipase (U/g protein)	54.53 ± 40.54	82.53 ± 22.57	72.18 ± 27.06	85.29 ± 11.61	82.95 ± 18.47	0.908	0.515	0.699
*α*‐amylase (U/mg protein)	0.12 ± 0.02	0.09 ± 0.04	0.12 ± 0.01	0.05 ± 0.00	0.10 ± 0.02	0.342	0.516	0.391
Cellulase (U/mg protein)	22.72 ± 1.38^c^	82.36 ± 7.93^a^	53.71 ± 0.46^b^	57.17 ± 8.10^b^	40.38 ± 11.64^bc^	0.002	0.756	0.045
Chitinase (U/g protein)	0.44 ± 0.11^ab^	0.81 ± 0.24^a^	0.16 ± 0.02^b^	0.48 ± 0.12^ab^	0.25 ± 0.01^b^	0.042	0.229	0.049
Sucrase (U/mg protein)	12.45 ± 2.80^c^	16.35 ± 4.30^bc^	36.52 ± 2.28^a^	40.23 ± 8.58^a^	29.28 ± 1.61^ab^	0.006	0.097	0.002
Maltase (U/mg protein)	9.55 ± 2.36^a^	15.03 ± 2.64^a^	10.26 ± 1.47^a^	21.31 ± 8.31^a^	39.38 ± 1.53^b^	0.003	<0.001	<0.001
Lactase (U/mg protein)	18.73 ± 11.48	13.25 ± 5.49	10.35 ± 1.88	10.21 ± 6.18	22.76 ± 4.96	0.630	0.467	0.248
Intestine								
Trypsin (U/mg protein)	1708.36 ± 255.75	2439.76 ± 223.73	1967.37 ± 315.08	1834.87 ± 287.02	1950.23 ± 285.69	0.445	0.834	0.968
Lipase (U/g protein)	36.66 ± 3.80^b^	65.17 ± 5.87^a^	56.88 ± 4.77^a^	38.69 ± 1.44^b^	32.74 ± 1.78^b^	0.001	0.067	0.044
*α*‐amylase (U/mg protein)	0.10 ± 0.02	0.08 ± 0.01	0.08 ± 0.01	0.07 ± 0.00	0.10 ± 0.01	0.191	0.546	0.059
Cellulase (U/mg protein)	12.46 ± 2.21	15.26 ± 1.19	17.79 ± 2.78	15.55 ± 5.16	15.14 ± 1.55	0.794	0.758	0.625
Chitinase (U/g protein)	0.13 ± 0.01	0.15 ± 0.01	0.13 ± 0.01	0.15 ± 0.00	0.15 ± 0.00	0.061	0.297	0.575
Sucrase (U/mg protein)	16.00 ± 1.50^a^	16.85 ± 1.13^a^	14.59 ± 2.25^a^	14.23 ± 2.76^a^	6.55 ± 2.28^b^	0.036	0.002	0.004
Maltase (U/mg protein)	24.69 ± 1.96	23.08 ± 7.97	15.24 ± 6.39	24.62 ± 2.79	27.23 ± 2.33	0.519	0.428	0.522
Lactase (U/mg protein)	1.76 ± 0.59	0.63 ± 0.09	2.21 ± 0.87	1.73 ± 0.13	2.90 ± 1.18	0.311	0.114	0.280
Esophagus								
Trypsin (U/mg protein)	3215.22 ± 393.49^ab^	3171.13 ± 487.13^ab^	3145.91 ± 68.39^ab^	3877.22 ± 255.22^a^	2310.87 ± 239.96^b^	0.044	0.153	0.040
Lipase (U/g protein)	311.13 ± 8.19^a^	312.05 ± 9.10^a^	203.17 ± 73.44^ab^	99.16 ± 10.31^bc^	73.53 ± 25.01^c^	0.001	<0.001	<0.001
*α*‐amylase (U/mg protein)	0.15 ± 0.03^a^	0.12 ± 0.01^ab^	0.14 ± 0.01^ab^	0.12 ± 0.01^ab^	0.09 ± 0.02^b^	0.034	0.013	0.041
Cellulase (U/mg protein)	0.02 ± 0.00^a^	0.02 ± 0.00^ab^	0.01 ± 0.00^b^	0.01 ± 0.00^b^	0.02 ± 0.00^ab^	0.024	0.614	0.005
Chitinase (U/g protein)	0.22 ± 0.01^ab^	0.21 ± 0.00^ab^	0.19 ± 0.00^ab^	0.19 ± 0.00^b^	0.23 ± 0.02^a^	0.024	0.447	0.023
Sucrase (U/mg protein)	37.25 ± 4.29^a^	16.11 ± 0.74^bc^	20.90 ± 1.16^b^	13.37 ± 0.05^c^	14.56 ± 0.74^bc^	<0.001	0.018	0.002
Maltase (U/mg protein)	25.68 ± 2.70^a^	21.73 ± 0.58^ab^	27.91 ± 1.22^a^	21.97 ± 2.27^ab^	15.21 ± 3.88^b^	0.035	0.009	0.023
Lactase (U/mg protein)	4.94 ± 1.38	1.55 ± 0.07	3.07 ± 2.06	1.68 ± 0.13	3.16 ± 0.40	0.274	0.662	0.269

*Note*: Data are presented as mean ± SE (*n* = 3). Values within the same row with different lowercase letters differ significantly (*p*  < 0.05). Linear and quadratic dose–response relationships were evaluated using orthogonal polynomial contrasts.

### 3.6. Intestinal Histology

Histological analysis revealed that chitin supplementation impacted intestinal integrity in a dose‐dependent and region‐specific manner (Table 8, Figure 1). Moderate supplementation (5%) improved foregut morphology, evidenced by significantly deeper crypts compared to the control. In contrast, high inclusion levels (10% and 20%) caused structural impairment, particularly in the midgut and hindgut. These groups exhibited significantly reduced muscle layer thickness, shorter and narrower villi, and a decrease in goblet cell abundance compared to the 5% group (*p*  < 0.05).

**Table 8:** Intestinal histology of juvenile *Ctenopharyngodon idella*.

Items	Diets					*p*‐Value		
	Diet 1	Diet 2	Diet 3	Diet 4	Diet 5	ANOVA	Linear	Quadratic
Foregut								
Villus number	26.83 ± 2.74	27.67 ± 0.88	30.00 ± 2.08	25.67 ± 1.20	26.33 ± 1.30	0.504	0.463	0.747
Muscle thickness (µm)	65.18 ± 4.52	60.14 ± 3.70	63.47 ± 1.98	56.34 ± 3.60	57.76 ± 1.06	0.322	0.112	0.186
Villus length (µm)	566.71 ± 29.44	625.81 ± 43.19	584.44 ± 73.22	484.23 ± 33.22	528.51 ± 53.21	0.364	0.212	0.376
Villus width (µm)	156.53 ± 4.37	154.47 ± 4.07	150.32 ± 22.98	154.62 ± 5.63	169.28 ± 4.19	0.789	0.275	0.404
Crypt depth (µm)	413.93 ± 14.17^a^	479.29 ± 36.08^ab^	520.17 ± 47.06^b^	450.97 ± 19.55^ab^	435.60 ± 24.94^ab^	0.016	0.687	0.035
Goblet cell number	18.42 ± 2.21^ab^	21.79 ± 1.01^a^	18.67 ± 2.30^ab^	17.58 ± 0.75^ab^	15.59 ± 0.79^b^	0.015	0.052	0.047
Midgut								
Villus number	20.17 ± 0.67	20.50 ± 2.57	25.67 ± 0.33	20.67 ± 3.17	20.17 ± 2.89	0.406	0.983	0.416
Muscle thickness (µm)	65.91 ± 1.59^a^	59.67 ± 0.32^bc^	62.50 ± 2.28^ab^	55.08 ± 2.77^c^	55.75 ± 1.38^c^	0.010	0.002	0.010
Villus length (µm)	570.04 ± 35.33	580.79 ± 43.76	569.40 ± 48.12	481.93 ± 26.66	487.91 ± 38.60	0.262	0.043	0.118
Villus width (µm)	126.77 ± 13.98	135.98 ± 8.99	136.07 ± 9.10	142.95 ± 5.56	109.41 ± 11.58	0.247	0.450	0.116
Crypt depth (µm)	316.66 ± 19.06	332.27 ± 21.75	352.19 ± 44.01	315.07 ± 12.38	287.89 ± 45.76	0.697	0.446	0.342
Goblet cell number	14.69 ± 0.43	15.90 ± 1.14	17.46 ± 4.16	13.40 ± 1.61	12.68 ± 1.58	0.567	0.348	0.325
Hindgut								
Villus number	16.83 ± 0.88^a^	24.67 ± 3.06^b^	25.00 ± 2.75^b^	26.17 ± 0.67^b^	20.00 ± 0.87^ab^	0.031	0.375	0.005
Muscle thickness (µm)	61.40 ± 5.04^a^	60.94 ± 5.60^a^	51.10 ± 4.21^ab^	45.63 ± 3.98^b^	42.69 ± 1.04^b^	0.033	0.001	0.005
Villus length (µm)	462.86 ± 31.11^ab^	568.17 ± 11.13^a^	521.59 ± 67.67^ab^	437.60 ± 9.63^b^	411.66 ± 35.54^b^	0.039	0.116	0.041
Villus width (µm)	141.88 ± 4.36^a^	139.26 ± 4.98^a^	144.30 ± 11.77^a^	115.17 ± 6.89^b^	118.26 ± 5.53^b^	0.001	0.002	0.006
Crypt depth (µm)	266.27 ± 67.73	313.25 ± 22.08	301.43 ± 41.79	261.84 ± 15.21	247.92 ± 23.03	0.266	0.263	0.127
Goblet cell number	15.81 ± 3.54^ab^	15.15 ± 2.69^ab^	18.79 ± 1.72^a^	13.98 ± 1.03^b^	12.90 ± 1.01^b^	0.041	0.179	0.035

*Note*: Data are presented as mean ± SE (*n* = 3). Values within the same row with different lowercase letters differ significantly (*p*  < 0.05). Linear and quadratic dose–response relationships were evaluated using orthogonal polynomial contrasts.

**Figure 1:**
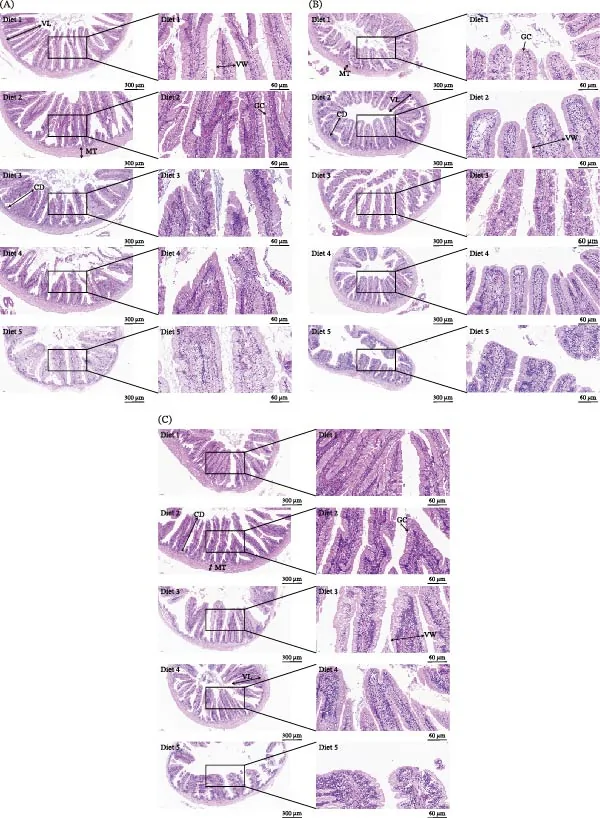
Intestinal histomorphology of juvenile grass carp fed diets containing different levels of *Periplaneta americana* chitin. (A) Foregut; (B) midgut; (C) hindgut. Within each panel, the five images on the left and the five images on the right were acquired at 10× and 30× magnification, respectively. MT, muscle thickness; VL, villus length; VW, villus width; CD, crypt depth; GC, goblet cells.

### 3.7. Gene Expression of Digestive Enzymes in the Intestine of Juvenile Grass Carp

Gene expression analysis (Figure 2) showed that the expression levels of trypsin, *α*‐amylase, and maltase were significantly lower in all chitin‐supplemented groups (Diets 2–5) than in the control group (Diet 1; *p*  < 0.05). In contrast, chitinase expression was significantly upregulated by dietary chitin, peaking in the 5% group (Diet 3) before decreasing at higher inclusion levels. Lactase expression was significantly lower in the 2.5% and 20% groups than in the control, 5%, and 10% groups (*p*  < 0.05), whereas intestinal lipase and sucrase expression did not differ significantly among treatments.

**Figure 2:**
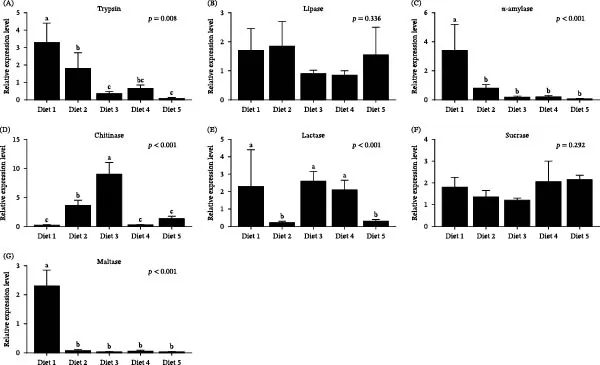
Relative mRNA expression of intestinal digestive‐enzyme genes in juvenile grass carp fed diets containing different levels of *Periplaneta americana* chitin. (A) Trypsin (*p* = 0.008); (B) lipase (*p* = 0.336); (C) *α*‐amylase (*p* < 0.001); (D) chitinase (*p* < 0.001); (E) lactase (*p* < 0.001); (F) sucrase (*p* = 0.292); and (G) maltase (*p* < 0.001). Data are presented as mean ± SE (*n* = 3 biological replicates). Different letters indicate significant differences among dietary groups (*p*  < 0.05). Expression levels were normalized to *β*‐actin. Because *β*‐actin Cq values differed among dietary treatments (F(4,10) = 8.21, *p* = 0.003), a sensitivity analysis based on raw target‐gene Cq values is provided in Supporting Information 1: Table S1.

Notably, the lower trypsin and *α*‐amylase transcript abundance in the 5% group was not accompanied by lower intestinal trypsin or *α*‐amylase activities (Table 7). This group simultaneously exhibited the highest crude‐protein ADC and favorable growth performance (Tables 3 and 4). Therefore, the endpoint transcriptional response did not indicate a reduction in the existing functional enzyme pool or in the integrated digestive performance of the fish.

### 3.8. Metabolomic Analysis of Gut Contents

The OPLS‐DA plot (Figure 3) showed a clear metabolic separation based on chitin dosage. The 2.5% (Diet 2) and 5% (Diet 3) groups clustered near the control (Diet 1), while the 10% (Diet 4) and 20% (Diet 5) groups formed distinct, separate clusters. The permutation test yielded a *Q* ^2^ intercept of −0.14 (Figure 4), indicating no substantial model overfitting based on the *Q* ^2^ criterion. Cluster analysis of differential metabolites (Figure 5) identified 17 key compounds. Taurodeoxycholic acid was elevated in the 10% and 20% groups. Conversely, 3,7,4^′^‐Trihydroxyflavone and pinostilbene were reduced in these high‐dose groups. KEGG pathway enrichment analysis (Figure 6, Table 9) identified 20 affected metabolic pathways. The most significantly impacted pathway across all groups was riboflavin metabolism (*p* < 0.001).

**Figure 3:**
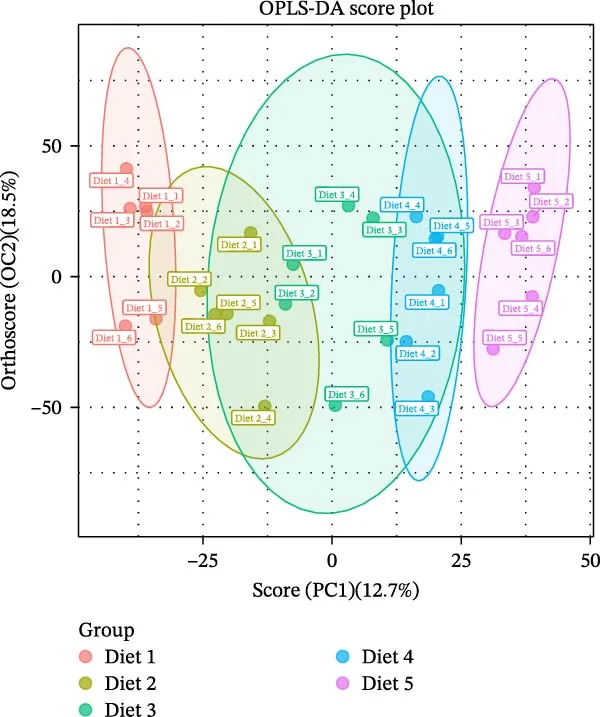
Orthogonal partial least squares discriminant analysis (OPLS‐DA) score plot of gut‐content metabolomic profiles in juvenile grass carp fed diets containing different levels of *Periplaneta americana* chitin.

**Figure 4:**
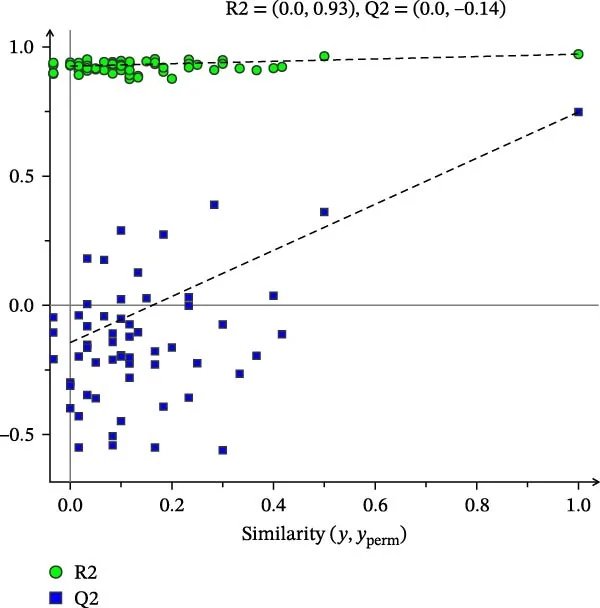
Permutation test for the OPLS‐DA model of gut‐content metabolomic profiles in juvenile grass carp.

**Figure 5:**
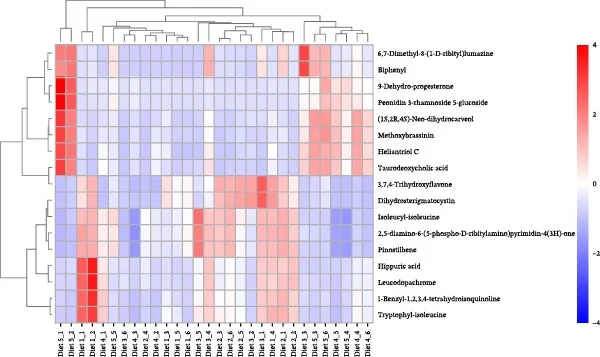
Clustering heatmap of 17 differential metabolites across the five dietary treatment groups. Columns represent individual samples, and rows represent differential metabolites. Hierarchical clustering was performed for both samples and metabolites, as indicated by the dendrograms at the top and left of the heatmap, respectively. The color scale represents the *Z*‐score‐standardized relative abundance of each metabolite, with red indicating higher abundance and blue indicating lower abundance.

**Figure 6:**
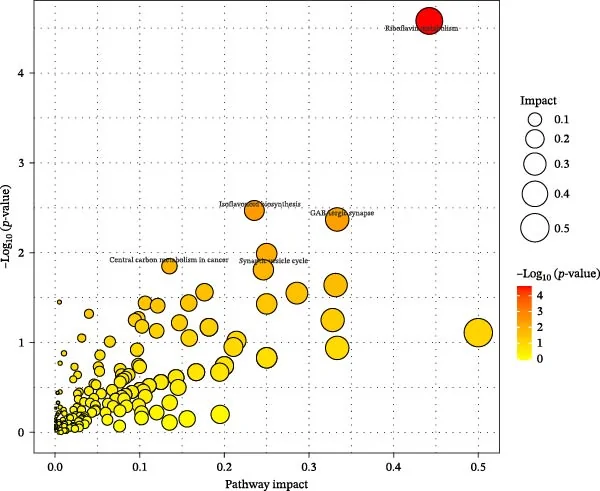
Pathway enrichment and topology analysis of metabolic pathways associated with differential metabolites. The *x*‐axis represents pathway impact values, and the *y*‐axis represents metabolic pathways. Bubble size indicates the number of differential metabolites mapped to each pathway, whereas bubble color represents the enrichment *p*‐value, with red indicating smaller *p*‐values and blue indicating larger *p*‐values.

**Table 9:** Metabolic pathways identified from significantly different metabolites in the gut of grass carp juveniles with different levels of *Periplaneta americana* chitin supplementation.

Metabolic pathway	*p*‐Value	Impact	Significantly different metabolites
Riboflavin metabolism	2.6 × 10^−5^	4.4 × 10^−1^	Flavin mononucleotide; D‐Ribulose 5‐phosphate; FOH 5_1; 6,7‐Dimethyl‐8‐(1‐D‐ribityl) lumazine; 5‐amino‐6‐ribitylamino uracil; 1‐deoxy‐L‐glycero‐tetrulose 4‐phosphate; 2,5‐diamino‐6‐(5‐phospho‐D‐ribitylamino) pyrimidin‐4(3 H)‐one
Isoflavonoid biosynthesis	3.4 × 10^−3^	2.4 × 10^−1^	Formononetin; genistein; genistin; ononin; 2^′^‐Hydroxygenistein; glyceollidin I; 9JHS2AVR43; 2‐Hydroxypseudobaptigenin
GABAergic synapse	4.3 × 10^−3^	3.3 × 10^−1^	L‐glutamic acid; L‐glutamine; γ‐aminobutyric acid
Synaptic vesicle cycle	1.0 × 10^−2^	2.5 × 10^−1^	L‐glutamic acid; γ‐aminobutyric acid; histamine
Central carbon metabolism in cancer	1.4 × 10^−2^	1.4 × 10^−1^	L‐glutamic acid; L‐glutamine; phosphoenolpyruvic acid; L‐histidine; isocitric acid
Stilbenoid, diarylheptanoid and gingerol biosynthesis	1.6 × 10^−2^	2.5 × 10^−1^	Chlorogenic acid; resveratrol; curcumin; demethoxycurcumin
Alanine, aspartate and glutamate metabolism	2.3 × 10^−2^	3.3 × 10^−1^	L‐glutamic acid; L‐glutamine; γ‐aminobutyric acid; aspartate
Proximal tubule bicarbonate reclamation	2.7 × 10^−2^	1.8 × 10^−1^	L‐glutamic acid; L‐glutamine; phosphoenolpyruvic acid
Nicotine addiction	2.8 × 10^−2^	2.9 × 10^−1^	L‐glutamic acid; γ‐aminobutyric acid
beta‐Alanine metabolism	3.6 × 10^−2^	5.1 × 10^−3^	L‐histidine; γ‐aminobutyric acid; pantothenic acid; beta‐Alanyl‐L‐arginine
Protein digestion and absorption	3.6 × 10^−2^	1.1 × 10^−1^	L‐glutamic acid; L‐glutamine; L‐histidine; histamine; isovaleric acid
Retrograde endocannabinoid signaling	3.7 × 10^−2^	1.6 × 10^−1^	L‐glutamic acid; glycerol; γ‐aminobutyric acid
Glutamatergic synapse	3.7 × 10^−2^	2.5 × 10^−1^	L‐glutamic acid; L‐glutamine
Taste transduction	3.9 × 10^−2^	1.2 × 10^−1^	L‐glutamic acid; alanine; γ‐aminobutyric acid; salicin
Carbon fixation in photosynthetic organisms	4.8 × 10^−2^	4.0 × 10^−2^	Phosphoenolpyruvic acid; D‐Ribulose 5‐phosphate; sedoheptulose 7‐phosphate
D‐Amino acid metabolism	5.4 × 10^−2^	9.8 × 10^−2^	L‐glutamic acid; L‐glutamine; alanine; L‐histidine; aspartate; D‐Alanyl‐D‐alanine
Neuroactive ligand–receptor interaction	5.6 × 10^−2^	9.4 × 10^−2^	L‐glutamic acid; γ‐aminobutyric acid; histamine; prostaglandin F2alpha; P‐hydroxyphenylethanolamine
Clavulanic acid biosynthesis	5.6 × 10^−2^	32.8 × 10^−2^	Deoxyamidinoproclavaminate; dihydroclavaminate
Arginine biosynthesis	6.0 × 10^−2^	14.7 × 10^−2^	L‐glutamic acid; L‐glutamine; N‐alpha‐acetylornithine
Diabetic cardiomyopathy	6.6 × 10^−2^	10.3 × 10^−2^	UDP‐α‐D‐N‐acetylglucosamine disodium; oxidized glutathione; Carnitine; glucosamine 1‐phosphate

*Note*: The table lists only the top 20 metabolic pathways ranked by significance level. The *p*‐value from the hypergeometric distribution test indicates that a smaller *p*‐value represents a more significant impact of the detected differential metabolites on the pathway. The impact value (Impact) represents the effect size of the metabolites on the pathway, with a larger value indicating a greater impact of the differential metabolites on the target pathway.

### 3.9. Gut Microbiota of Juvenile Grass Carp

#### 3.9.1. Functional Characteristics of the Gut Microbiota (Metagenomics)

To investigate the functional potential of the gut microbiota, shotgun metagenomic sequencing was performed. Metabolism was the predominant first‐level functional category (Figure 7), with amino acid metabolism being the most abundant second‐level category (Figure 8). A cluster heatmap of the top 20 functional genes (Figure 9) revealed distinct functional profiles. The 5% group (Diet 3) was uniquely enriched in genes related to protein synthesis, including ribosomal proteins K02518, K02950, and K02992. In contrast, the 10% group (Diet 4) showed the highest abundance of stress‐related genes, including the cold‐shock protein gene K03704, and nitrogen metabolism genes, including glutamine synthetase K01915.

**Figure 7:**
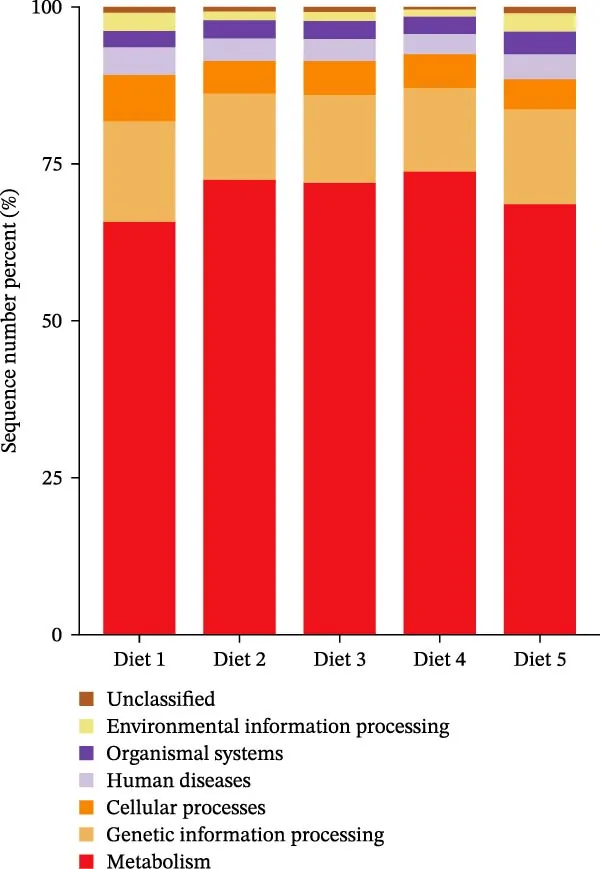
KEGG Level 1 functional profiles of the gut microbiota in juvenile grass carp fed diets containing different levels of *Periplaneta americana* chitin. Bars represent the relative abundances of functional categories across the five dietary treatment groups.

**Figure 8:**
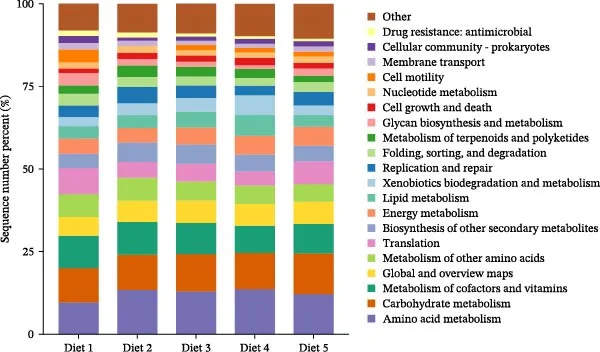
KEGG Level 2 functional profiles of the gut microbiota in juvenile grass carp fed diets containing different levels of *Periplaneta americana* chitin. Bars represent the relative abundances of functional categories across the five dietary treatment groups.

**Figure 9:**
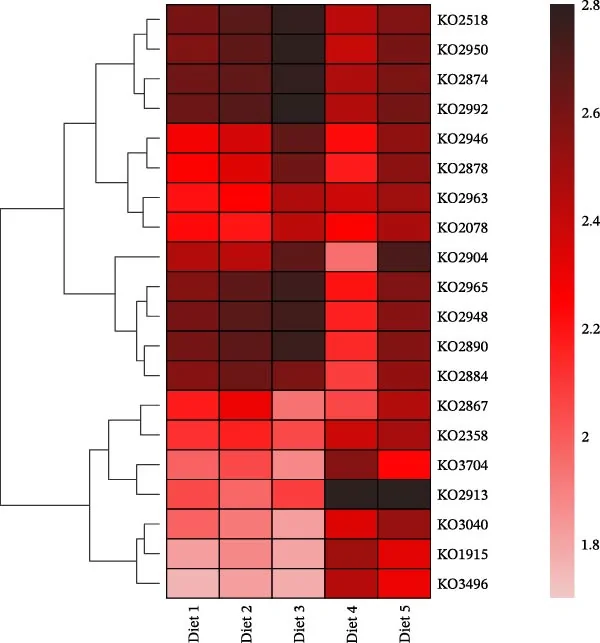
Clustering heatmap of the 20 most abundant KEGG Orthology (KO) entries in the gut microbiota of juvenile grass carp across the five dietary treatment groups. Rows represent KO entries, and columns represent dietary groups. Color intensity indicates relative abundance according to the scale shown.

The metabolomic analysis identified riboflavin metabolism as the most significantly enriched pathway (*p* < 0.001). We therefore re‐examined the metagenomic data for microbial riboflavin‐biosynthesis potential. Neither the riboflavin metabolism pathway (ko00740; *F* (4,10) = 0.28, *p* = 0.883) nor the bacterial riboflavin‐biosynthesis module M00125 (F(4,10) = 0.36, *p* = 0.834) differed significantly among diets. Among the individual biosynthesis genes, only ribA (K01497; GTP cyclohydrolase II) was significantly reduced after FDR correction (F(4,10) = 7.78, *p* = 0.004, FDR *p* = 0.041; Tukey HSD: Diet 1 vs. Diets 2–4, *p*  < 0.05). ribB was nominally significant (*p* = 0.014) but did not remain significant after FDR (FDR *p* = 0.069), while ribD, ribE, ribH, ribF, and ribBA showed no significant differences (*p* > 0.05). At the tank level, *Cetobacterium* relative abundance was negatively but nonsignificantly correlated with M00125 abundance (Spearman *ρ* = − 0.382, *p* = 0.160). Inspection of the taxon‐stratified KO table showed no *Cetobacterium*‐associated reads carrying riboflavin‐biosynthesis KOs; the module‐level signal was contributed mainly by unclassified bacteria and Aeromonas (Supporting Information 2: Figure S1 and Supporting Information 3: Table S2).

#### 3.9.2. Composition and Diversity of the Gut Microbiota in Juvenile Grass Carp

16S rRNA amplicon sequencing identified 78,653 ASVs across all groups (Figure 10). Alpha diversity analysis (Figure 11) showed that the 10% group (Diet 4) exhibited the highest species richness and diversity (Chao1, Shannon, etc.). Beta diversity analysis (Figure 12) showed that the 10% (Diet 4) and 20% (Diet 5) groups were clearly separated from the control, 2.5%, and 5% groups in the PCoA ordination. At the phylum level (Figure 13), the high‐dose groups (Diets 4 and 5) saw a dramatic rise in *Pseudomonadota* and a collapse of *Fusobacteriota*. At the genus level (Figure 14), *Cetobacterium* was dominant in the low‐dose groups (Diets 1–3), whereas its relative abundance was markedly reduced in the high‐dose groups (Diets 4–5). This reduction was accompanied by significant increases in the relative abundances of *Cupriavidus* and *Neisseria*.

**Figure 10:**
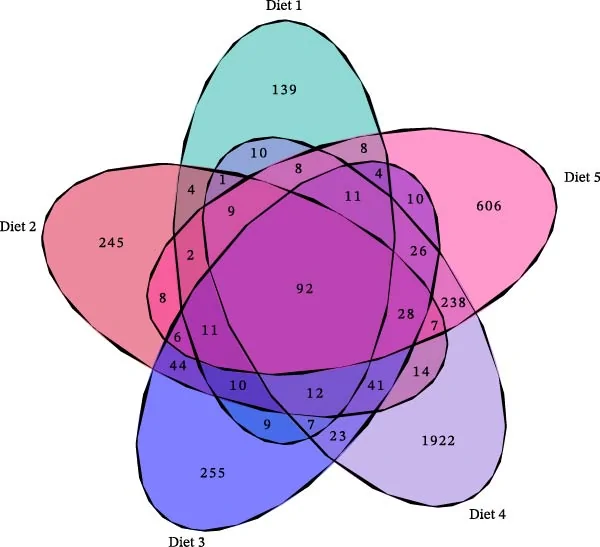
Venn diagram showing shared and unique amplicon sequence variants (ASVs) of the gut microbiota among the five dietary treatment groups. Each ellipse represents one dietary group (Diet 1–Diet 5), and the numbers indicate ASVs unique to individual groups or shared among overlapping groups.

**Figure 11:**
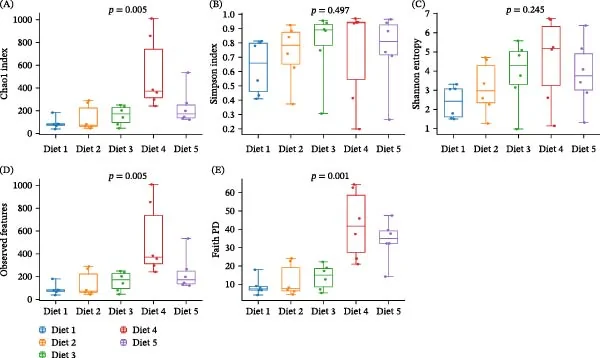
Alpha‐diversity indices of the gut microbiota in juvenile grass carp across the five dietary treatment groups. (A) Chao1 index; (B) Simpson index; (C) Shannon entropy; (D) observed features; and (E) Faith’s phylogenetic diversity (Faith PD). Individual points represent biological samples. *p*‐values indicate overall differences among dietary groups based on the Kruskal–Wallis test.

**Figure 12:**
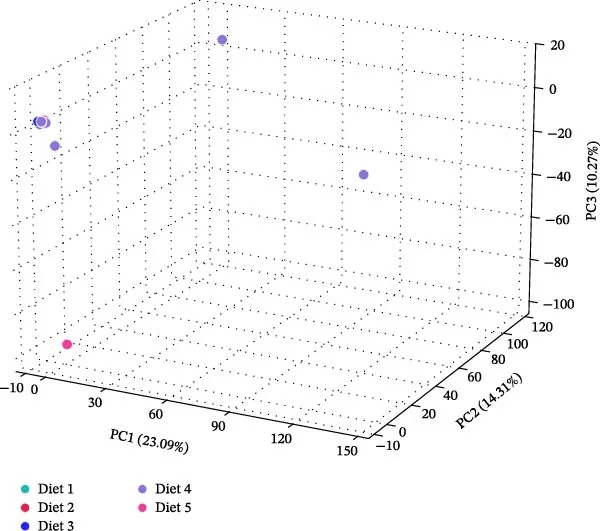
Three‐dimensional principal coordinates analysis (PCoA) plot showing the beta‐diversity pattern of gut microbial communities in juvenile grass carp fed diets containing different levels of *Periplaneta americana* chitin.

**Figure 13:**
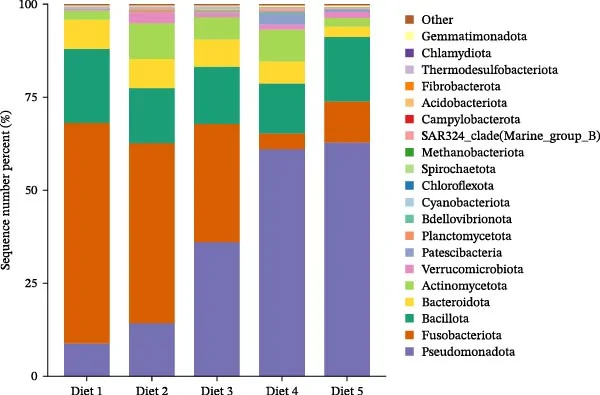
Relative abundance of dominant microbial phyla in the gut of juvenile grass carp across the five dietary treatment groups. The top 20 phyla are shown, and the remaining taxa are grouped as “Other”.

**Figure 14:**
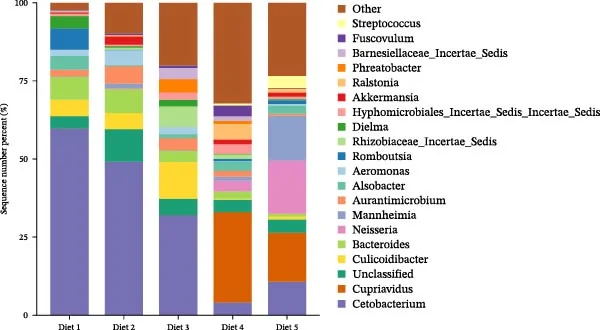
Relative abundance of dominant microbial genera in the gut of juvenile grass carp across the five dietary treatment groups. The top 20 genera are shown, and the remaining taxa are grouped as “Other”.

### 3.10. Correlation Between Major Gut Metabolites and Microbiota

Spearman’s correlation analysis (Figure 15) was performed to link microbial shifts to metabolic changes. The analysis based on amplicon data (Figure 15A) revealed strong correlations. Genera within the Pseudomonadota phylum, such as *Vibrio* and *Shewanella*, showed strong positive correlations with metabolites like 1‐Hydroxypyrene and strong negative correlations with L‐Glutamine. The analysis based on metagenomic data (Figure 15B) further confirmed significant interdependencies between specific microbial taxa and metabolites, such as the negative correlation between *Desulfobulbia* and LysoPC (0_0_18_0).

**Figure 15:**
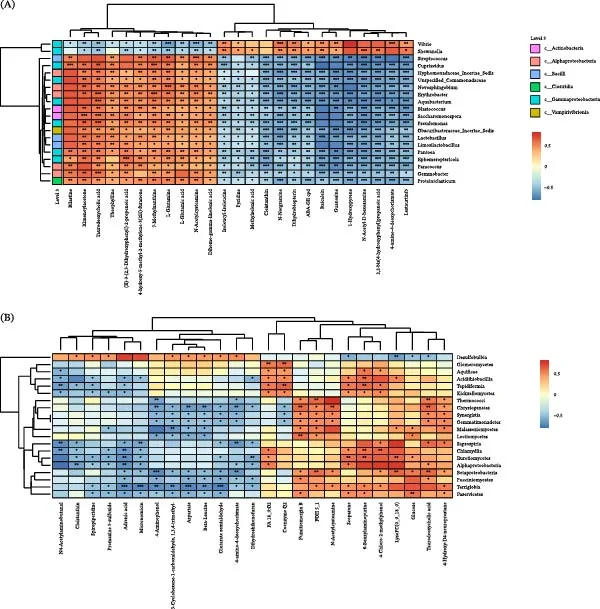
Correlations between gut metabolites and microbial taxa in juvenile grass carp. (A) Correlations based on amplicon‐sequencing data. (B) Correlations based on metagenomic‐sequencing data. Red and blue indicate positive and negative correlations, respectively, with color intensity representing correlation strength. Statistical significance is indicated as follows:  ^∗∗∗^ *p*  < 0.001;  ^∗∗^ 0.001 ≤ *p*  < 0.01; and  ^∗^ 0.01 ≤ *p*  < 0.05.

Although these correlations demonstrate coordinated changes in microbial taxa and intestinal metabolites, they do not establish the direction or causality of the relationships. In particular, the correlations between *Cetobacterium*, microbial functional potential, and riboflavin‐related metabolites cannot by themselves demonstrate that the depletion of this genus directly altered riboflavin synthesis.

## 4. Discussion

### 4.1. Dose‐Dependent Effects of Dietary Chitin on Growth and Nutrient Utilization

The central hypothesis of this study was that dietary *P. americana* chitin would exert a strong, dose‐dependent effect on juvenile grass carp. Our results confirm this hypothesis, revealing a distinct optimal inclusion window. Among the tested inclusion levels, 5% produced the most favorable overall response, including the highest growth performance, crude‐protein digestibility, and intestinal chitinase transcript abundance. This aligns with broader observations that insect‐derived functional ingredients, when used at appropriate levels, can positively modulate fish physiology [31]. Conversely, high inclusion levels (10% and 20%) were clearly detrimental, suppressing growth, impairing intestinal integrity, and accompanied by marked restructuring of the gut microbial community. This biphasic dose–response is a critical finding for the practical application of this novel chitin source in aquaculture.

The primary indicators of feed efficacy, growth performance, and feed utilization clearly delineated this optimal dose. The 5% chitin group (Diet 3) exhibited the highest FBW, WGR, and SGR, together with the lowest FCR and the highest PER. This favorable growth response was accompanied by improved nutrient utilization: the ADC of crude protein peaked in the 5% group (Diet 3) at 74.34%, which was significantly higher than both the control (72.01%) and all higher‐dose groups. This result suggests that the improved growth performance at the 5% inclusion level was at least partly associated with enhanced protein utilization. In addition, the 5% group exhibited the highest intestinal chitinase transcript abundance and relatively high chitin digestibility, indicating that juvenile grass carp retained some capacity to respond adaptively to a moderate dietary chitin load [32].

The significant quadratic responses of growth and nutrient‐utilization indices indicate that the 63‐day feeding period was sufficient to detect the physiological effects of dietary chitin under the present experimental conditions. However, the sharp decline in growth observed in the 10% and 20% groups demonstrates that chitin, at high concentrations, acts as a potent antinutritional factor. This is consistent with previous findings where high levels of crustacean‐derived chitin (10%–25%) inhibited growth in species like tilapia and rainbow trout [18, 19]. Taken together, these results indicate that 5% was the most favorable inclusion level among those tested, whereas inclusion levels of 10% or higher adversely affected feed utilization and growth.

### 4.2. Reconciling Digestive‐Enzyme Transcription With Digestive Performance

A notable finding of this study was that trypsin, *α*‐amylase, and maltase transcript levels were reduced in the 5% chitin group despite its superior growth performance and crude‐protein digestibility. This apparent inconsistency does not necessarily represent a direct biological contradiction, because transcript abundance, biochemical enzyme activity, and apparent digestibility represent different aspects and temporal scales of digestive physiology in the present study. RT‐qPCR measured mRNA abundance at a single endpoint after the 24 h fasting period, whereas the biochemical assays reflected enzyme activity in intestinal tissues at the time of sampling. In contrast, the ADC integrated digestive and absorptive performance over the 28‐day fecal‐collection period. Thus, these measurements represented an endpoint transcriptional response, intestinal enzyme activity at sampling, and long‐term in vivo nutrient utilization, respectively. Consequently, endpoint mRNA abundance should not be interpreted as a direct surrogate for either enzyme activity or long‐term nutrient digestibility.

At the 5% inclusion level, reduced trypsin and *α*‐amylase transcript abundance was not accompanied by lower intestinal trypsin or *α*‐amylase activities. Thus, the lower mRNA levels did not translate into a measurable reduction in the existing functional enzyme pool. Several processes may have contributed to this transcription–activity uncoupling, including continued translation from pre‐existing transcripts, post‐transcriptional buffering, substrate‐associated stabilization or reduced degradation of previously synthesized enzymes, relatively slow enzyme turnover, and compensatory maintenance of enzyme activity. The moderate dietary chitin load may also have induced an early adaptive digestive response, after which transcription declined through feedback regulation while sufficient enzyme protein and activity were retained. Because digestive‐enzyme protein abundance, translation efficiency, protein half‐life, postprandial expression kinetics, and luminal enzyme activity were not directly measured, these explanations should be regarded as biologically plausible interpretations rather than experimentally demonstrated mechanisms.

In addition, *α*‐amylase and maltase primarily participate in carbohydrate digestion and therefore cannot directly account for the higher crude‐protein ADC in the 5% group. Although trypsin transcript abundance was reduced, intestinal trypsin activity remained statistically unchanged. Meanwhile, the 5% group exhibited the highest intestinal chitinase expression, relatively high chitin digestibility, maintained intestinal *α*‐amylase activity, favorable intestinal morphology, and maintained or increased activities of intestinal lipase and sucrase compared with the high‐dose groups. Taken together, these responses indicate that the overall digestive and absorptive capacity was preserved or improved at the moderate inclusion level despite the reduced expression of several individual digestive‐enzyme genes. Therefore, the improved growth and protein digestibility in the 5% group are better explained by the integrated digestive phenotype than by the expression pattern of any single digestive‐enzyme gene. The physical‐restriction hypothesis is not used to explain the response of the 5% group but is considered specifically in relation to the impaired digestive performance observed at the excessive 10% and 20% inclusion levels.

### 4.3. Possible Physical Restriction of Digestion at High Chitin Inclusion Levels

The observed growth inhibition at high doses was accompanied by a marked reduction in nutrient digestibility. The ADC of crude protein peaked in the 5% group but decreased sharply in the 10% and 20% groups to values significantly below that of the control. Chitin digestibility showed a similar dose‐dependent decline at the two highest inclusion levels. These results indicate that juvenile grass carp were able to utilize a moderate dietary chitin load but that excessive inclusion was associated with a substantial decline in their capacity to digest and utilize this structural polysaccharide.

The physical‐restriction hypothesis is considered specifically in relation to the impaired digestive performance observed in the 10% and 20% groups and is not intended to explain the improved protein digestibility in the 5% group. At high inclusion levels, excessive dietary chitin may increase digesta bulk, reduce the accessibility of nutrients to digestive enzymes, or restrict effective enzyme–substrate contact within the intestinal lumen [32, 33]. These effects could reduce nutrient hydrolysis and absorption and thereby contribute to the lower crude‐protein ADC and growth performance. However, the present study did not directly assess digesta viscosity, nutrient coating, chitin–nutrient interactions, or enzyme–substrate accessibility. Therefore, physical restriction should be considered a plausible contributing process rather than a demonstrated causal mechanism. Moreover, the unchanged tissue enzyme activities cannot be taken as evidence that homogenization disrupted a chitin matrix and restored enzyme–substrate contact, because this possibility was not directly examined.

The intestinal histological findings provide additional evidence that the digestive and absorptive environment was adversely affected at high chitin inclusion levels. Histological analysis revealed significant and regionally specific structural alterations in the 10% and 20% groups, particularly in the midgut and hindgut. These fish displayed significantly thinner muscle layers and reduced villus height and width. These structural alterations may have reduced the effective absorptive surface and contributed to the lower nutrient digestibility and growth performance. Furthermore, the number of goblet cells, which contribute to mucus secretion and maintenance of the mucosal barrier [34], was significantly reduced in the foregut and hindgut of the high‐dose groups. This reduction suggests weakened mucosal protection, although intestinal permeability, inflammatory responses, and pathogen translocation were not directly assessed in the present study.

The high‐dose groups also exhibited changes in several circulating metabolic indicators, including serum AST, glucose, and triglyceride levels. These responses are consistent with altered systemic metabolism under excessive chitin inclusion but should not, in isolation, be interpreted as direct evidence of liver injury or pathological dysfunction [35, 36]. Taken together, the concurrent reductions in feed intake and nutrient digestibility, together with the intestinal structural alterations, suggest that excessive dietary chitin impaired nutrient acquisition through a combination of reduced nutrient accessibility and a less favorable intestinal absorptive environment. Nevertheless, the relative contributions of these processes could not be determined from the present experimental design. Direct characterization of digesta physicochemical properties, nutrient accessibility, and in vivo enzyme–substrate interactions will be required to validate the proposed physical‐restriction mechanism.

### 4.4. Microbiome‐Associated Alterations in Riboflavin Metabolism

The gut microbial community exhibited a marked dose‐dependent response to dietary *P. americana* chitin. The control, 2.5%, and 5% groups maintained a microbial community dominated by *Cetobacterium*, whereas the 10% and 20% groups showed a pronounced reduction in this genus together with an increased relative abundance of Pseudomonadota. *Cetobacterium* is commonly detected as a major intestinal bacterium in freshwater fish and has been associated with nutrient metabolism and the production of B‐group vitamins [37–39]. However, the metabolic contribution of *Cetobacterium* may vary with bacterial strain, host species, diet composition, and interactions with other members of the microbial community. Therefore, its reduced abundance in the high‐dose groups cannot, by itself, be considered direct evidence of impaired microbial vitamin synthesis. The increased abundance of Pseudomonadota further indicates substantial restructuring of the intestinal microbial community, consistent with the broader evidence that fish gut microbiota can be markedly influenced by dietary and environmental factors [39].

The 10% group exhibited the highest alpha diversity. In the context of the marked reduction in the dominant genus *Cetobacterium* and the clear separation of the high‐dose groups in the beta‐diversity analysis, this increase in diversity may reflect microbial community restructuring rather than an unequivocal improvement in intestinal health. Because community stability, temporal succession, and resistance to disturbance were not directly evaluated, the biological significance of the increased alpha diversity should be interpreted cautiously.

The functional profiles of the gut microbiota also differed among dietary treatments. The 5% group was enriched in several genes associated with microbial protein synthesis, including ribosomal‐protein genes, suggesting relatively active microbial biosynthetic potential under moderate chitin supplementation. In contrast, the 10% group showed relatively high abundances of stress‐response and nitrogen‐metabolism genes, including a cold‐shock protein gene and glutamine synthetase. These changes indicate that the microbial community underwent functional adjustment in response to the intestinal conditions associated with high‐dose chitin. The functional alterations occurred concurrently with reduced nutrient digestibility and intestinal structural changes. However, the present experimental design cannot determine whether the microbial changes contributed to the impaired intestinal condition or arose secondarily from changes in nutrient availability, intestinal structure, or host physiology.

The metabolomic profiles of the 10% and 20% groups were clearly separated from those of the control and 5% groups, and riboflavin metabolism was identified as the most significantly altered metabolic pathway. To further evaluate the potential functional relationship between the microbial changes and riboflavin‐related metabolism, the metagenomic dataset was reanalyzed for genes assigned to the KEGG riboflavin metabolism pathway (ko00740) and the bacterial riboflavin‐biosynthesis module (M00125), including *ribA*, *ribB*, *ribD*, *ribE*, and *ribH*. The targeted analysis showed that no significant differences were detected in the annotated riboflavin‐biosynthesis pathway or module among the dietary groups, and only ribA (K01497) was significantly reduced after FDR correction (F(4,10) = 7.78, *p* = 0.004, FDR *p* = 0.041; Tukey HSD: Diet 1 vs. Diets 2–4, *p*  < 0.05). The abundance of the microbial riboflavin‐biosynthesis module was not significantly correlated with the relative abundance of *Cetobacterium* (Spearman’s *ρ* = − 0.382, *p* = 0.160).

Taxon‐stratified analysis of the HUMAnN results showed that *Cetobacterium*‐associated sequences contributed none of the annotated riboflavin‐biosynthesis potential in any dietary group; the module‐level signal was instead attributable mainly to unclassified bacteria, Aeromonas in the control group, and Aurantimicrobium and the *Bacillus cereus* group in the 2.5% and 5% groups. Consistent with this, intestinal riboflavin concentrations themselves did not differ significantly among diets (*p* = 0.62), whereas the immediate biosynthetic precursor 6,7‐dimethyl‐8‐ribityllumazine showed a tendency to accumulate with increasing dietary chitin (*p* = 0.009, FDR *p* = 0.067). These observations argue against a simple vitamin B2‐deficiency model and instead suggest that the metabolomic enrichment of riboflavin metabolism reflects broader alterations in riboflavin‐related metabolism rather than a specific loss of microbial biosynthetic capacity.

The correlation analysis additionally demonstrated coordinated changes between bacterial taxa and intestinal metabolites. Genera within Pseudomonadota, including *Vibrio* and *Shewanella*, were positively associated with 1‐hydroxypyrene and negatively associated with L‐glutamine, whereas other microbial taxa were correlated with lipid‐related metabolites. These associations may reflect microbial metabolic activity, changes in substrate availability, host metabolic responses, or shared responses of the microbiota and metabolome to the altered intestinal environment. Therefore, the correlations identify coordinated microbial and metabolic variation but do not establish the direction of the relationships.

Taken together, the results indicate that the depletion of *Cetobacterium* was accompanied by alterations in riboflavin‐related metabolism without clear functional‐gene evidence of reduced microbial biosynthetic potential. Nevertheless, the parallel taxonomic, functional, and metabolomic changes do not establish a direct causal pathway. Other microbial taxa may contribute substantially to the detected riboflavin‐biosynthesis genes, and both the microbial and metabolic alterations may have arisen as parallel responses to the intestinal environmental changes induced by high‐dose chitin.

Furthermore, KEGG pathway enrichment indicates that the differential metabolites were statistically associated with riboflavin metabolism but does not directly demonstrate reduced microbial riboflavin production or physiological vitamin B2 deficiency in the host. Accordingly, the present findings are interpreted as evidence of microbiome‐associated alterations in riboflavin‐related metabolism rather than confirmation of a causal microbiota–riboflavin metabolic axis. Direct validation will require targeted quantification of riboflavin, flavin mononucleotide, and flavin adenine dinucleotide, together with taxon‐resolved functional analyses, microbial isolation, *Cetobacterium* supplementation, or other controlled intervention experiments.

### 4.5. Integrated Interpretation and Study Limitations

Taken together, the physiological and multiomics data support an integrated dose‐dependent interpretation of the effects of dietary *P. americana* chitin in juvenile grass carp. At the 5% inclusion level, intestinal digestive‐enzyme activities were maintained, crude‐protein digestibility and growth performance were improved, intestinal morphology remained generally favorable, and the core microbial community was largely preserved. In contrast, the 10% and 20% inclusion levels were associated with reduced feed intake, impaired nutrient digestibility, intestinal structural alterations, depletion of *Cetobacterium*, expansion of Pseudomonadota, and changes in riboflavin‐related metabolism. These findings suggest that restricted nutrient utilization and microbiome‐associated metabolic alterations may coexist at excessive chitin inclusion levels. However, their relative contributions and interactions could not be directly quantified in the present study.

Several limitations should be acknowledged. First, digestive‐enzyme protein abundance, translation efficiency, protein turnover, and in vivo luminal enzyme activities were not measured. Therefore, the biological processes underlying the observed uncoupling between digestive‐enzyme transcript abundance and physiological performance remain uncertain. Second, the proposed physical restriction of digestion was not directly evaluated through measurements of digesta viscosity, nutrient coating, chitin–nutrient interactions, or enzyme–substrate accessibility. Accordingly, physical inhibition should be regarded as a plausible contributing process rather than a confirmed mechanism. Third, the associations among microbial taxa, riboflavin‐biosynthesis functional genes, and intestinal metabolites were derived from observational multiomics analyses and therefore do not establish causality. In particular, the concurrent depletion of *Cetobacterium* and alteration of riboflavin‐related metabolism do not demonstrate that the loss of this genus directly reduced microbial riboflavin synthesis or caused vitamin B2 deficiency in the host. Moreover, the enrichment or alteration of the KEGG riboflavin metabolism pathway reflects changes in riboflavin‐related metabolic profiles but cannot be interpreted as direct evidence of a physiological riboflavin deficiency. Fourth, *β*‐actin was the sole reference gene used for RT‐qPCR normalization and showed a significant between‐diet Cq shift (*p* = 0.003). Although aquarium‐level analyses of raw target‐gene Cq values reproduced the principal treatment effects, this sensitivity analysis does not demonstrate reference‐gene invariance. The qPCR findings should therefore be interpreted with appropriate caution, and future studies should validate multiple reference genes under the experimental conditions used.

Future studies should quantify digestive‐enzyme protein abundance and luminal activity and characterize the physicochemical properties of intestinal digesta. Targeted quantification of riboflavin, flavin mononucleotide, and flavin adenine dinucleotide, together with microbial isolation, *Cetobacterium* supplementation, and other targeted functional‐validation experiments, will be required to determine whether specific microbial taxa directly contribute to riboflavin‐related metabolism and associated host physiological responses. Despite these limitations, the consistent biphasic responses observed across growth, nutrient digestibility, intestinal morphology, digestive physiology, microbial composition, and metabolomic profiles provide a practical basis for the dietary application of *P. americana* chitin. Under the conditions of this study, 5% represented the most favorable inclusion level, whereas inclusion levels of 10% or higher were associated with impaired nutrient utilization and intestinal homeostasis.

## 5. Conclusion

Dietary supplementation with *P. americana* chitin exerted dose‐dependent effects in juvenile grass carp. An inclusion level of 5% significantly increased weight gain, SGR, and ADC of crude protein. However, inclusion levels of 10% and 20% impaired nutrient utilization and intestinal condition and were accompanied by marked restructuring of the gut microbial community, including a pronounced reduction in *Cetobacterium* and an increase in Pseudomonadota. Metagenomic reanalysis further showed that the riboflavin‐biosynthesis gene ribA was significantly reduced at intermediate chitin doses, although overall microbial riboflavin‐biosynthesis potential and intestinal riboflavin levels were maintained, suggesting alterations in riboflavin‐related metabolism rather than a frank vitamin B2 deficiency. However, the present findings do not establish that *Cetobacterium* depletion directly caused reduced riboflavin synthesis or host vitamin B2 deficiency. Among the inclusion levels tested, 5% produced the most favorable overall response under the present experimental conditions.

## Author Contributions

Xiaofang Zou conducted the investigation and formal analysis, and wrote the original draft. Qiujie Chen and Wei Pu contributed to the methodology, investigation, and formal analysis. Jingjing Gao participated in data curation, methodology, and investigation. Yongshou Yang helped with visualization and methodology. Xiaowen Long conceptualized the study, supervised the project, acquired funding, and reviewed and edited the manuscript.

## Funding

This study was funded by the Dali University Doctoral Research Startup Fee Project (Grant KYBS2021064); the Yunnan Fundamental Research Projects (Grant 202501AT070431); the Yunnan Province “Xingdian Talent Support Plan” Young Talents Special Project (Grant 230212524080); and the Dali University Scientific Research Development Fund Special Project (Grant KY2519104940).

## Disclosure

A preprint of this study has previously been published [40]. All authors contributed to the article and approved the submitted version.

## Conflicts of Interest

The authors declare no conflicts of interest.

## Supporting Information

Additional supporting information can be found online in the Supporting Information section.

## Supporting information


**Supporting Information 1** Table S1: Quantification cycle (Cq) values of β‐actin across the five dietary treatments and evaluation of its expression stability.

**Supporting Information 2** Figure S1: Abundances of riboflavin‐biosynthesis genes and module M00125 across dietary treatments, and the association between *Cetobacterium* relative abundance and M00125 abundance.

**Supporting Information 3** Table S2: Relative abundances of the riboflavin metabolism pathway, riboflavin‐biosynthesis module, and associated genes across dietary treatments.

## Data Availability

The data that support the findings of this study are available from the corresponding author upon reasonable request.
